# High intrinsic phase stability of ultrathin 2M WS_2_

**DOI:** 10.1038/s41467-024-45676-3

**Published:** 2024-02-10

**Authors:** Xiangye Liu, Pingting Zhang, Shiyao Wang, Yuqiang Fang, Penghui Wu, Yue Xiang, Jipeng Chen, Chendong Zhao, Xian Zhang, Wei Zhao, Junjie Wang, Fuqiang Huang, Cao Guan

**Affiliations:** 1 Institute of Flexible Electronics, Northwestern Polytechnical University, Xi’an, 710072 China; 2 Key Laboratory of Flexible Electronics of Zhejiang Province, Ningbo Institute of Northwestern Polytechnical University, 218 Qingyi Road, Ningbo, 315103 China; 3 State Key Laboratory of Solidification Processing, Northwestern Polytechnical University, Xi’an, 710072 Shaanxi China; 4 State Key Laboratory of High-Performance Ceramics and Superfine Microstructure, Shanghai Institute of Ceramics, Chinese Academy of Sciences Shanghai, Shanghai, 200050 China; 5 Qian Xuesen Laboratory of Space Technology, China Academy of Space Technology, Beijing, 100094 China

**Keywords:** Two-dimensional materials, Two-dimensional materials

## Abstract

Metallic 2M or 1T′-phase transition metal dichalcogenides (TMDs) attract increasing interests owing to their fascinating physicochemical properties, such as superconductivity, optical nonlinearity, and enhanced electrochemical activity. However, these TMDs are metastable and tend to transform to the thermodynamically stable 2H phase. In this study, through systematic investigation and theoretical simulation of phase change of 2M WS_2_, we demonstrate that ultrathin 2M WS_2_ has significantly higher intrinsic thermal stabilities than the bulk counterparts. The 2M-to-2H phase transition temperature increases from 120 °C to 210 °C in the air as thickness of WS_2_ is reduced from bulk to bilayer. Monolayered 1T′ WS_2_ can withstand temperatures up to 350 °C in the air before being oxidized, and up to 450 °C in argon atmosphere before transforming to 1H phase. The higher stability of thinner 2M WS_2_ is attributed to stiffened intralayer bonds, enhanced thermal conductivity and higher average barrier per layer during the layer(s)-by-layer(s) phase transition process. The observed high intrinsic phase stability can expand the practical applications of ultrathin 2M TMDs.

## Abstract (web summary)

Metallic 2M or 1T′-phase transition metal dichalcogenides (TMDs) are characterized by interesting properties, but they are metastable. Here, the authors demonstrate that the thermal and oxidation stability of 2M WS_2_ significantly increases as the thickness is reduced down to monolayer, expanding the potential applications of 2M TMDs.

## Introduction

Phase engineering of group-VI transition metal dichalcogenides (TMDs) such as MoS_2_ and WS_2_ is important for acquiring novel physical and chemical properties^1–6^. Depending on coordination modes between the transition metal and chalcogen atoms, these TMDs present in either trigonal prismatic coordinated semiconductive phases (2H and 3R) or octahedral coordinated metallic phases (2M, T_d_, 1T, 1T′, etc.)^1–3,6^. Bulk 2M and the monolayered (ML) 1T′ WS_2_ have shown plenty of unique appealing properties, such as superconductivity^7–10^, Weyl semimetal states^11,12^, optical nonlinearity^13,14^ and enhanced electrochemical activities^15–19^. However, the 1T′ TMDs are metastable and tend to transform to the thermodynamically stable 2H phase^1,3,4^, hence their practical applications are significantly limited^1,3^. Although various strategies such as electron doping^20,21^, strain effect^22,23^, and heterostructural interaction^24,25^ can be adopted to stabilize the metallic phase TMDs, their intrinsic stabilities under practical operation conditions (e.g., temperature and atmosphere) remain unclear.

As van der Waals (vdW)-bonded layered materials, physicochemical properties of TMDs can dramatically change when their structures evolve from three-dimensional bulk to ultrathin two-dimensional (2D) regime. For example, band gaps of 2H MoS_2_ and WS_2_ turn from indirect to direct after thickness is reduced to monolayer^26^. Dielectric screening in gapped 2D crystals significantly reduces with decrease of layer number, leading to greatly enhanced Coulomb interactions^27^. The high Coulombic interactions have resulted in tightly bonded exitons and trions in ML MoS_2_^27^, as well as anormal thickness-dependent Raman shifts^28^. Atomically thin TMDs exhibit much higher electrocatalytic activities than bulk matrixes, because of “self-gating effect” induced high carrier densities^29,30^. The in-plane inversion symmetry of bulk TMDs is broken after they are reduced to ML, which produces out-of-plane spin polarization depending on the valley (K or −K point) in momentum space^2^. Thermal conductivity is found to decrease with increase of layer number for thin 2H MoS_2_ and T_d_ WTe_2_^31,32^, while the opposite trend is seen for thin indium selenides^33,34^. In a word, the layer number plays critical roles in both the electronic and phonon properties of 2D TMDs. As 2M to 2H phase transition of WS_2_ (or 1T′ to 1H for ML WS_2_) involves changes in energy band structures of both electron and phonon, the layer number could greatly affect the stability of 2M WS_2_. However, the relation between layer number and stability of 2M WS_2_ is barely known to the best of our knowledge.

Here, we investigate the thickness-dependent intrinsic phase stability of mechanically exfoliated 2M WS_2_ and find that thinner samples have higher thermal stabilities. 2M to 2H phase transition temperature increases from 120 °C to 210 °C in the air as thickness of WS_2_ is reduced from bulk to bilayer (2L). ML WS_2_ can maintain 1T′ structure in the air and argon (Ar) atmosphere until temperature reaches 350 °C and 450 °C, respectively, which are about two and three times higher than phase transition temperatures of bulk 2M WS_2_. Raman spectroscopy reveals thinner 2M WS_2_ has more stiffened intralayer W‒W and W‒S bonds and higher thermal conductance, which enables thinner samples with higher resistance to lattice deformation and higher efficiency heat dissipation under elevated temperature. Theoretical simulation and calculation further confirm thinner WS_2_ has a higher average energy barrier per layer during the layer(s)-by-layer(s) phase transition process.

## Results

### Thickness-dependent phase stability of 2M WS_2_

**Fig. 1:**
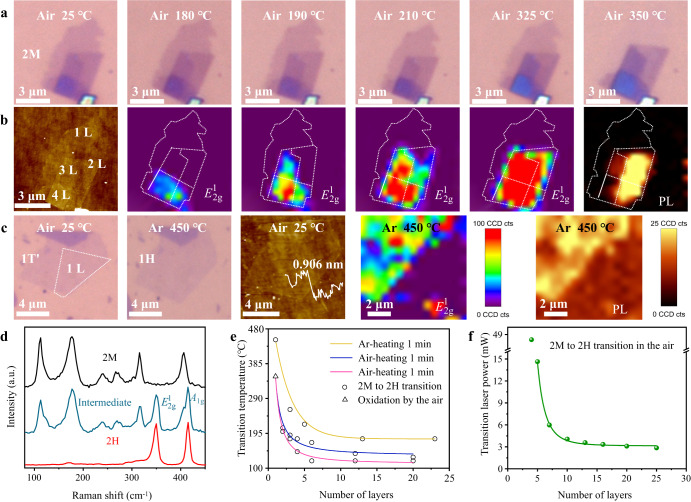
Thickness-dependent phase stability of 2M WS_2_ on Si/SiO_2_ substrate. **a** Optical and **b** atomic force microscope (AFM) images and mappings of *E*^1^_2g_ Raman mode and photoluminescence (PL) intensities of a piece of exfoliated 2M WS_2_ flake with different thicknesses areas at different temperatures. AFM image is measured at room temperature, and the corresponding height profile is shown in Supplementary Fig. 3f. **c** Optical images, AFM image, and mappings of *E*^1^_2g_ Raman mode and PL intensities of a piece of exfoliated monolayered (ML) 1T′ WS_2_ flake at room temperature in the air and after heated at 450 °C in Ar atmosphere. Mappings measured area is outlined by dotted lines in the optical image. All the scale bars in (**a**–**c**) correspond to 2 μm. **d** Raman spectra of a 2M WS_2_ and an intermediate phase and 2H WS_2_ that were obtained by heating multilayered 2M WS_2_ at 120 °C for 5 min and at 250 °C for 20 min, respectively. **e** 2M to 2H (or 1T′ to 1H) phase transition temperatures (circle points) and air oxidation temperature (triangle point) as functions of WS_2_ layer thickness, measured in the air or Ar atmosphere with temperature elevated by 5 °C and held for 1 min or 15 min in each step. Solid lines are guides to the eye. **f** The power of laser required to activate 2M to 2H phase transition as a function of WS_2_ layer thickness measured in the air. A solid line is a guide to the eye.

2M WS_2_ is synthesized through solid-state chemical method as reported previously^7^. The as-synthesized 2M WS_2_ shows distinctive Raman peaks at 110.9, 117.1, 177.2, 241.7, 268.6, 316.3, and 406.5 cm^−1^ (black line in Fig. 1d), the same as previously reported results^3,7^. Rietveld refinement based on powder X-ray diffraction (XRD) (Supplementary Fig. 1 and Supplementary Table 1) reveals the synthesized WS_2_ has monoclinic structure in *C*_2/m_ space group with cell parameters *a* = 12.8417 Å, *b* = 3.2177 Å, *c* = 5.6912 Å and *β* = 112.8368°, which is in excellent agreement with the previously reported 2M WS_2_ structure^3,7^. X-ray photoelectron spectroscopy (XPS) (Supplementary Fig. 2) further evidences the synthesized sample is composed of only W and S elements. All these results confirm the high purity of the synthesized 2M WS_2_. Once 2M to 2H phase change is triggered by heat or oxidation, two additional Raman peaks appear at around 350.2 and 420.0 cm^−1^ (blue line in Fig. 1d) that are attributed to the *A*_1g_ and ${E}_{2{{\mbox{g}}}}^{1}$ vibration modes of 2H WS_2_^3^. After the WS_2_ flake is completely converted to 2H phase, all the 2M characteristic vibration modes disappear and only the *A*_1g_ and ${E}_{2{{\mbox{g}}}}^{1}$ peaks can be seen (red line in Fig. 1d). Accordingly, the intensity of ${E}_{2{{\mbox{g}}}}^{1}$ mode can be used as an indicator for the extent of phase change within a 2M WS_2_ flake.

Here, we define phase transition temperature as the one at which the 2M or 1T′ WS_2_ starts to lose phase purity, i.e., the ${E}_{2{{\mbox{g}}}}^{1}$ Raman mode emerges, or the WS_2_ sample begins to be oxidized by the air. To investigate the thickness-dependent phase stability, 2M WS_2_ flakes are mechanically exfoliated into thin layers. Figure 1a,  b shows optical and atomic force microscope (AFM) images of a piece of exfoliated 2M WS_2_ flake with thickness varying from ML to 4L. This sample was treated with stepped heating in the air with temperature elevated by 5 °C and held for 1 min in each stage, during which optical images and mappings of ${E}_{2{{\mbox{g}}}}^{1}$ Raman mode and photoluminescence (PL) intensities are acquired to address the location of 2M to 2H (or 1T′ to 1H) phase change, as shown in Fig. 1a, b. It is found that the ${E}_{2{{\mbox{g}}}}^{1}$ mode can be observed at the 4L area when temperature goes up to 180 °C, whereas it does not appear at the 3L and 2L areas until the temperature goes to 190 °C, 210 °C, respectively. With temperature increasing from 180 °C to 325 °C, the ${E}_{2{{\mbox{g}}}}^{1}$ mode intensities of 3–4L areas keep increasing and the transparency is obviously decreasing, while the 2L area is too thin to tell transparency change. It is known that 2H WS_2_ has higher refractive index and lower transparency than 2M WS_2_^35^. PL peaks (Supplementary Fig. 3a–c) at ~640 nm emerge in 2–4L areas as temperature reaches 325 °C, further confirming the 2M to 2H phase transitions^36^. The PL intensities are significantly enhanced after heating to 350 °C, as shown in Supplementary Fig. 3a–c, consistent with the increase of ${E}_{2{{\mbox{g}}}}^{1}$ mode intensity at elevated temperature. These results indicate 2M to 2H phase transition is a slow process, and the extent of phase transition augments at elevated temperatures or after longer time of heating. PL intensity of WS_2_ is sensitive to thickness^26^, and the distribution of PL intensity (Fig. 1b) agrees to the AFM defined areas of various thicknesses from 2L to 4L. Comparatively, the ML WS_2_ area preserves the typical 1T′ Raman pattern without emergence of ${E}_{2{{\mbox{g}}}}^{1}$ mode or PL until temperature reaches 350 °C (Fig. 1b and Supplementary Fig. 3d, e), evidencing the highest thermal stability among the four WS_2_ areas. In agreement with this result, chemically exfoliated ML 1T′ WS_2_ is also demonstrated to have good air-stability at room temperature^37,38^. Heated at 350 °C in the air, Raman signal of the ML 1T′ WS_2_ fades away and the ML 1T′ WS_2_ flake disappears, seen from Fig. 1a, which can be attributed to decomposition due to air oxidation. In our experiments of heating in the air, we never observe oxidation of 2L or multilayered 2M WS_2_ flakes before 2M to 2H phase transition, while we never observe 1T′ to 1H phase transition on a ML 1T′ WS_2_ flake before it was oxidized and decomposed. This means 1T′ to 1H phase transition temperature of ML 1T′ WS_2_ is higher than the air oxidation and decomposition temperature. After moving to an inert Ar atmosphere, 1T′ to 1H phase transition temperature of ML WS_2_ (Fig. 1c) is measured to be as high as 450 °C, confirmed by the reduced transparency and emergences of ${E}_{2{{\mbox{g}}}}^{1}$ mode and PL (Fig. 1c). These results strongly indicate thinner 2M WS_2_ has higher phase stability.

To further confirm the thickness-dependent stability of 2M WS_2_, we have prepared exfoliated samples with thickness covering a wide range (from 23L to ML) and systematically investigated their tolerance to heat in the air or Ar atmosphere. The optical, AFM images and Raman spectra of these samples at representative conditions can be found in Supplementary Figs. 4–6. Figure 1e summarizes phase transition temperatures of the investigated 2M WS_2_ samples during stepped heating programs. When heating in the air with temperature held for 1 min in each stage as aforementioned, the measured phase transition temperature continuously increases from 120 °C to 350 °C as WS_2_ is thinned from 20L to ML (blue line in Fig. 1e). When temperature is held for 15 min in each stage, the phase transition temperatures of thicker samples (≥6L) converge to a same level of 120 °C, while the phase transition temperatures of thinner samples (1‒5L) still maintain the thickness dependence (red line in Fig. 1e). Regardless of temperature holding time, ultrathin samples (ML and 2L) exhibit significantly high phase transition temperatures that are over 200 °C. When heating in Ar atmosphere, the same trend of phase transition temperature as function of sample thickness is also observed, except all 2M (or 1T′) WS_2_ samples show increased phase transition temperatures compared with heating in the air (yellow line in Fig. 1e). Previous X-ray absorption fine structure measurements have confirmed enrichment of electrons on 2M or 1T′ WS_2_ surface^3,6^. It has also been demonstrated that donation of electrons helps to stabilize 1T′ TMDs, while extraction of electrons promotes 1T′ to 1H phase transition^20,21,39–41^. Thus, higher 2M to 2H (or 1T′ to 1H) phase transition temperature measured in the inert atmosphere can be attributed to getting rid of electron acceptors, such as O_2_ and H_2_O molecules in the air. High-resolution XPS (Supplementary Fig. 7) shows that W 4f, S 2p and valance band edge spectra of 2M WS_2_ shift to higher energies after 2M to 2H phase transition is activated by heating in the air, and higher extent of phase transition leads to larger range of shifting. Meanwhile, no peak attributed to W‒O or S‒O chemical bond can be found in the W 4 f and S 2p spectra and O 1s spectra (probably contributed by adsorbed or intercalated H_2_O and O_2_ molecules) barely change during the 2M to 2H phase transition. These results indicate air speeds up 2M to 2H phase transition during heating processes by extracting electrons from 2M WS_2_ without chemically oxidizing it. The thinner 2M WS_2_ can also withstand higher intensity incident laser during Raman measurements (Fig. 1f and Supplementary Fig. 8). From 25L to 5L WS_2_, the intensity of laser required to activate the 2M to 2H phase transition increases from 2.8 mW to 14.6 mW. A 48.3 mW laser, which is the maximum output of the used instrument, can turn a 4L WS_2_ to 2H phase, but is not able to activate the phase transition for 1‒3L 2M WS_2_ samples.

### 2M to 2H phase transition mechanism

**Fig. 2:**
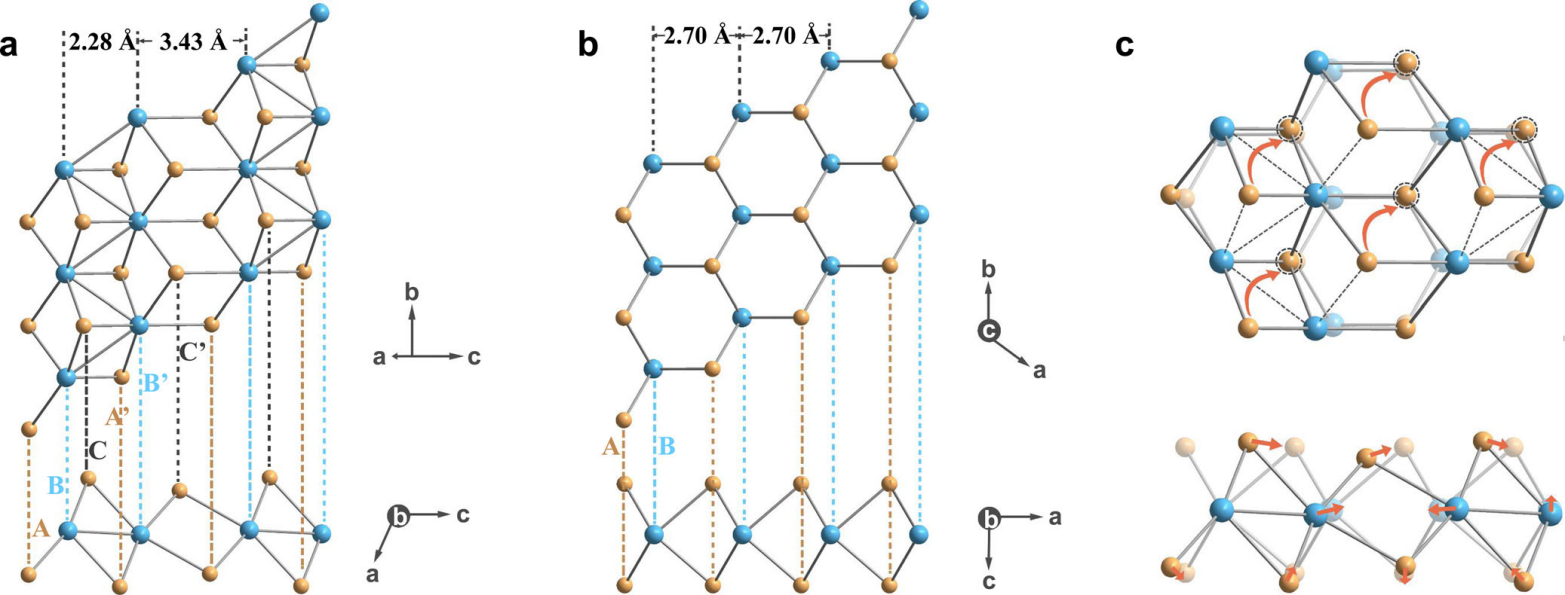
2M to 2H phase transition mechanism of WS_2_. **a** Crystal structures of 1T′ WS_2_, where W and S atoms coordinate in an octahedral configuration and stack in an A/A’-B/B’-C/C’ mode. **b** Crystal structures of 1H WS_2_, where W and S atoms coordinate in a trigonal prismatic configuration and stack in an A-B-A mode. **c** Top view (upper) and side view (lower) of crystal lattices when overlapping of a layer of 1T′ WS_2_ on top of a layer of 1H WS_2_. Color code: blue and orange spheres represent W and S, respectively. The broken W‒W and W‒S bonds are labeled by dash lines, and the directions of W and S atoms displacements are indicated by orange arrows. Each S atom that dissociates from a W atom after breaking the W‒S bond moves to a new position (marked by a dotted line circle) and forms a new W‒S bond.

To understand how WS_2_ transforms from 2M to 2H phase, we closely investigate crystal structures of the two phases. Each layer of 2M (or a layer of 1T′) WS_2_ contains distorted [WS_6_]^8−^ octahedrons sharing edges along *bc* plane, and W atoms form W–W zigzag chains along *b* direction, resulting in nonuniform distances between W-atom lines, *d*_1_ = 2.28 Å and *d*_2_ = 3.43 Å (Fig. 2a and Supplementary Fig. 9a)^7^. Viewing from the *b* direction, W and S atoms reside in six different planes and stack in an A/A’-B/B’-C/C’ model (Fig. 2a)^7^. Every two neighbored S atoms located in different planes (A and C or A’ and C’) share two W atoms (Supplementary Fig. 9a). Comparatively, each layer of 2H (or a layer of 1H) WS_2_ is composed of standard [WS_6_]^8−^ trigonal prisms with uniform W-atom line distance of *d*_3_ = 2.70 Å (Fig. 2b and Supplementary Fig. 9b)^42^. Viewing from the *b* direction, W and S atoms stack in a simple A-B-A sandwiched model (Fig. 2b), and every two neighbored S atoms located in different planes share three W atoms (Supplementary Fig. 9b)^42^.

Because of the structural differences between 2M and 2H WS_2_, the 2M to 2H phase change involves reforming of intralayer bond network with displacements of W and S atoms and changing of interlayer spacing. As shown in Fig. 2c, during 2M to 2H phase change of WS_2_, the bonded W‒W chains break up and W atoms in these chains gradually separate along *c* direction. The gliding of W atoms finally gives uniform W-atom line distance, approaching that in 2H WS_2_. In the meantime, S atoms residing in C and C’ planes glide along the *bc* direction to lay on top of adjacent S atoms in A and A’ planes, respectively. Subsequently, each S atom dissociates from the initially bonded W atom and forms a new covalent bond with another adjacent W atom, resulting in alteration of W–S coordination from octahedron to trigonal prism, as shown in Fig. 2c. Along with out-of-plane displacements of W and S atoms and adjusting of W–S bond lengths and W–S–W angles, each [WS_6_]^8−^ layer changes from the six-plane stack (2M) to the three-plane sandwiched stack (2H) (Fig. 2c). Since 2H WS_2_ has smaller average W‒S bond length, W–S–W bond angle and W-atom line distance but larger interlayer spacing than the 2M WS_2_ (Supplementary Fig. 9), 2M to 2H phase transition experiences slight in-plane contraction and vertical expansion.

**Fig. 3:**
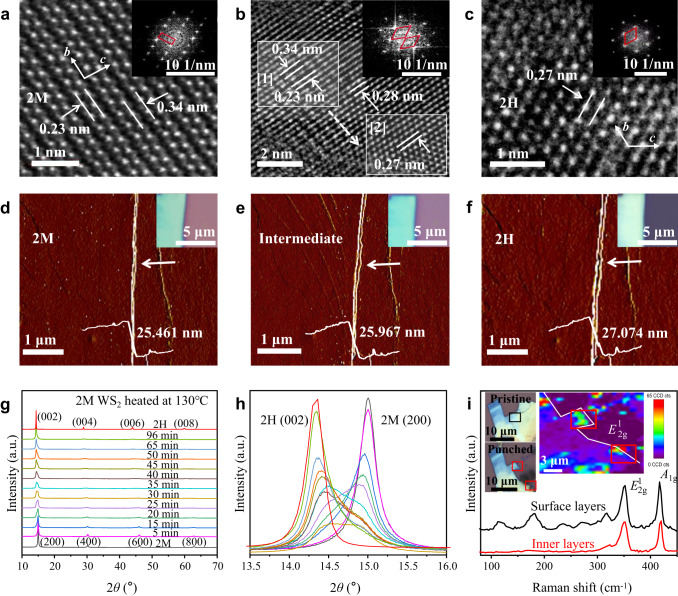
Structural characterization of WS_2_ during 2M to 2H phase transition. High-resolution transmission electron microscopy (HRTEM) images and the corresponding Fourier transforms of (**a**) 2M and (**b**) an intermediate phase WS_2_. **c** A zoomed-in view and Fourier transform of area [2] in (**b**). **d** Powder X-ray diffraction (XRD) patterns of 2M, intermediate phases and 2H WS_2_. 2H and intermediate phases WS_2_ were obtained by heating 2M WS_2_ in the air at 250 °C for 20 min and at 130 °C for various times, respectively. **e** A zoomed-in view of the XRD patterns in the range of 13.5° ~ 16.0°. AFM images with height profiles and optical images (insets at top-right corners) of a piece of multilayered WS_2_ flake in (**f**) 2M, (**g**) an intermediate and (**h**) 2H phases. Edge of the WS_2_ flake is denoted by white arrow, where interlayer sliding can be seen. **i** Raman spectra of an intermediate phase multilayered WS_2_ flake measured from the surface layers (black line) and the exposed inner layers (red lines) after punching with a tungsten probe. Insets show the corresponding optical images of the pristine WS_2_ flake and the one after punching (left) and Raman ${E}_{2{{\mbox{g}}}}^{1}$ mode intensity mapping of the probe-punched WS_2_ flake (right). Raman-measured areas are defined by squares.

The slowness of 2M to 2H phase transition of WS_2_ allows us to trace this process by investigating structures of intermediate phases after varied extents of 2M to 2H phase change. Intermediate phases WS_2_ can be obtained by heating the 2M WS_2_ above phase transition temperature within controlled time. As shown in Supplementary Figs. 4–6, all intermediate phases WS_2_ flakes show superpositions of 2M and 2H characteristic Raman modes, indicating the intermediate phases of WS_2_ are probably 1T′/1H heterostructures that can involve in-plane heterostructured areas and hybrid stacked layers. Figure 3a–c show high-resolution transmission electron microscopy (HRTEM) images of W-atom configurations in 2M and an intermediate phase. Zigzag chains of W atoms are observed in Fig. 3a, in consistency with the reported structure of 2M WS_2_^3^. Area [1] of the intermediate phase WS_2_ (Fig. 3b) reveals W-atom lines distances ranging from 0.26 to 0.31 nm, which are in between *d*_1_ (0.23 nm) and *d*_2_ (0.34 nm) of 2M WS_2_ (Fig. 3a). Fourier transform of Area [1] displays irregular quadrilaterals (inset of Fig. 3b) that are distorted from the rectangular pattern of 2M WS_2_ (inset of Fig. 3a). Area [2] of the intermediate phase WS_2_ has a W-atom lines distance of 0.27 nm (Fig. 3c) and hexagonal pattern Fourier transform (inset of Fig. 3c), which are the same as 2H WS_2_^42^. These results demonstrate that an intermediate phase WS_2_ has an in-plane heterostructure of distorted 1T′ and 1H, which is formed due to gliding of W atoms.

To investigate layer stacking configurations of intermediate phases WS_2_, AFM images are taken for different phases WS_2_. It is observed that the thickness of a WS_2_ flake (Fig. 3d–f) increases from 25.461 nm to 25.967 nm and 27.074 nm as it changes from 2M to an intermediate and 2H phase, revealing the continuous change of interlayer spacing. Moreover, there is slight sliding at edge of the WS_2_ flake from 2M to the intermediate and 2H phase (Fig. 3d–f and Supplementary Fig. 12), indicating generation of interlayer dislocation during the phase change. Since transforming from a 1T′ to 1H layer results in in-plane lattice contraction, the interlayer dislocation can be attributed to non-concerted 1T′ to 1H phase transition among different layers and the followed reconfiguration of layer stacking. XRD has been carried out on bulk 2M WS_2_ powder during heating at 130 °C. With prolongation of heating time, XRD peaks attributed to 2M (200) diffractions are weakening and broadening, while the ones attributed to 2H (002) diffractions are intensifying and sharpening, and all these peaks are shifting to lower degrees but locating in between that of standard 2M (200) and 2H (002) diffractions, as shown in Fig. 3g, h. These results indicate ordered stacks of 1T′ and 1H layers are broken and built, respectively, with increase of phase change extent. An intermediate phase WS_2_ has hybrid low-order stacks of 1T′ and 1H layers with average interlayer spacing in between that of pure 2M and 2H phase. In agreement with this result, Raman spectra (Supplementary Fig. 10) of intermediate phases WS_2_ manifests that *A*_1g_ mode (ascribed to out-of-plane vibration of 1H layer stacks) locates at lower wave number compared to 2H phase and keeps to blue-shift as the extent of 2M to 2H phase change increases. High-index XRD diffractions including 2M $(11\bar{1})$ (002) $(31\bar{1})$ (601) (311) are also disappearing with $(11\bar{1})$ diffraction shifting to higher degrees as heating time increases, as shown in Supplementary Fig. 11. This can be attributed to in-plane gliding and out-of-plane displacements of W and S atoms during the 2M to 2H phase transition process. The above XRD, Raman and AFM results hint that 2M to 2H phase transition of multilayered WS_2_ probably takes place layer(s) by layer(s). To further confirm this mechanism, we tested Raman spectra on an intermediate phase multilayered WS_2_ flake before and after punching with a tungsten probe. As shown in Fig. 3i, the exposed inner layers after punching (areas marked by red squares) exhibit significantly higher intensities of 2H *A*_1g_ and ${E}_{2{{\mbox{g}}}}^{1}$ modes than top layers of the WS_2_ flake, which strongly evidences non-concerted phase transition among different layers.

### Thickness-dependent thermal properties of 2M WS_2_

**Fig. 4:**
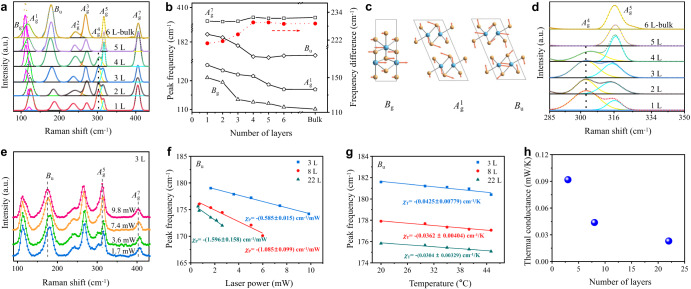
Thickness-dependent intralayer bond strength and thermal conductance of 2M WS_2_ on Si/SiO_2_ substrate. **a** Raman spectra of exfoliated thin (1 ~ 6L) and bulk 2M WS_2_. The observed active Raman modes are labeled beside the corresponding peaks. **b** Frequencies of *B*_g_, ${A}_{{{\mbox{g}}}}^{1}$, *B*_u_ and ${A}_{{{\mbox{g}}}}^{7}$ Raman modes and the difference between frequencies of *B*_u_ and ${A}_{{{\mbox{g}}}}^{7}$ modes (red dotted line) as a function of layer thickness of 2M WS_2_. **c** Atomic displacements of the *B*_g_, ${A}_{{{\mbox{g}}}}^{1}$ and *B*_u_ Raman modes. *B*_g_ mode is viewed in the *bc* plane, while ${A}_{{{\mbox{g}}}}^{1}$ and *B*_u_ modes are viewed in the *ac* plane. **d** ${A}_{{{\mbox{g}}}}^{4}$ and ${A}_{{{\mbox{g}}}}^{5}$ Raman peaks of exfoliated thin (1–6L) and bulk 2M WS_2_. **e** Raman spectra of a 3L 2M WS_2_ acquired with different power laser excitation. Frequency of *B*_u_ mode as functions of **f** laser power and **g** temperature for 3L, 8L and 22L 2M WS_2_. Laser-power coefficient (${\chi }_{{{\mbox{P}}}}$) and temperature coefficient (${\chi }_{{{\mbox{T}}}}$) of B_u_ mode frequencies are extracted from slopes of the corresponding plots. **h** Thermal conductivities of 3L, 8L and 22L 2M WS_2_ estimated by ${\chi }_{{{\mbox{T}}}}$ divided by ${\chi }_{{{\mbox{P}}}}$. For each sample, an average thermal conductance is obtained based on three values calculated from three sets of ${\chi }_{{{\mbox{T}}}}$ and ${\chi }_{{{\mbox{P}}}}$ data that are extracted from *B*_u_ mode plots in (**f**, **g**) and ${A}_{{{\mbox{g}}}}^{5}$ and ${A}_{{{\mbox{g}}}}^{7}$ modes plots in Supplementary Figs. 14, 15.

As the 2M to 2H phase change of WS_2_ occurs along with dissociations of W–W bonds and reconstructions of W–S bonds, strengths of these intralayer chemical bonds play a critical role in phase stability of 2M WS_2_. Characteristic vibration frequency ($\sigma$) of a chemical bond is in proportion to square root of the bond force constant ($k$), according to $\sigma=({N}_{A}^{0.5}/2\pi ){(k/M)}^{0.5}$, where *N*_*A*_ is Avogadro’s constant, and *M* is average atomic mass. Therefore, frequencies of Raman vibration modes of 2M WS_2_ manifest strengths of the intralayer chemical bonds. Figure 4a shows Raman spectra of 2M WS_2_ flakes with various thicknesses. All samples exhibit the same Raman pattern, indicating the exfoliated few-layered 2M WS_2_ flakes maintain the same distorted octahedral coordination as that of bulk one. Previous theoretical calculation has demonstrated 2M WS_2_ has nine active Raman vibration modes, including one *B*_g_ mode, one *B*_u_ mode and seven *A*_g_ modes (${A}_{{{\mbox{g}}}}^{6}$ at 327.5 cm^−1^ is not detectable in our results), as denoted in Fig. 4a^3^. Interestingly, *B*_g_, ${A}_{{{\mbox{g}}}}^{1}$ and *B*_u_ modes are found to distinctly stiffen (blue shift) with decrease of thickness. As shown in Fig. 4b, from 6L to ML 1T′ WS_2_, *B*_g_ mode shifts from 110.9 to 121.1 cm^−1^, with ${A}_{{{\mbox{g}}}}^{1}$ mode shifting from 117.1 to 125.5 cm^−1^ and *B*_u_ mode shifting from 177.2 to 184.7 cm^−1^. For flakes of 6 or more layers, the frequencies of these modes converge to the bulk values. Other Raman modes of ML 1T′ WS_2_ were also reported to blue shift compared with that of bulk 2M one^3^, but we are not able to see these shifts in our results, probably because the shifts are too small to be observed. As shown in Fig. 4c, *B*_g_ mode corresponds to in-plane gliding of W and S atoms, while ${A}_{{{\mbox{g}}}}^{1}$ mode corresponds to distortion of W‒S bonds and *B*_u_ mode corresponds to stretching W–W bonds, along with out-of-plane displacements of S atoms^3^. Stiffening of Raman vibration modes with decrease of layer number indicates increases in W–W and W‒S bond force constants, leading to more rigid S–W–S lattices in thinner 2M WS_2_. This accounts for the thickness-dependent stability of 2M WS_2_. It should be noted that the high stability of ultrathin 2M WS_2_ cannot be ascribed to substrate effect. The same trends of thickness-dependent Raman mode frequencies and phase stability of 2M WS_2_ are also observed when the substrate is changed from Si/SiO_2_ to polydimethylsiloxane (PDMS) that has minimum interaction with TMD flakes, as shown in Supplementary Fig. 13.

Additionally, the different thickness dependence of different Raman modes allows Raman frequency to be an indicator of the layer thickness. We have depicted the difference (Δ*ω*) between frequencies of *B*_u_ and ${A}_{{{\mbox{g}}}}^{7}$ modes in Fig. 4b. For ML WS_2_, Δ*ω* is 220.5 cm^−1^, which is smaller than the value of 221.5 cm^−1^ for 2L and 224.5 cm^−1^ for 3L WS_2_. From ML to bulk WS_2_, the Δω increases from 220.5 cm^−1^ to 229.2 cm^−1^. This statistic of Raman mode frequencies provides a powerful tool for rapidly identifying thickness of 2M TMD, similar to that used for analyzing thickness of 2H TMD^28^. Another useful measure is the ${A}_{{{\mbox{g}}}}^{4}$ mode that turns to be detectable when 2M WS_2_ is thinned to 4L as shown in Fig. 4d. ${A}_{{{\mbox{g}}}}^{4}$ mode has an intensity comparable with ${A}_{{{\mbox{g}}}}^{5}$ mode in 3L 2M WS_2_ and exhibits higher intensity than ${A}_{{{\mbox{g}}}}^{5}$ mode in 2L 2M WS_2_. Moreover, ${A}_{{{\mbox{g}}}}^{4}$ intensity is two times that of ${A}_{{{\mbox{g}}}}^{5}$ mode in ML 1T′ WS_2_ (Fig. 4d). The enhancing of ${A}_{{{\mbox{g}}}}^{4}$ mode with decreasing of layer number probably associates with change of vibration symmetries in ultrathin WS_2_^2^.

As 2M to 2H phase transition of multilayered WS_2_ is probably a layer(s)-by-layer(s) slow process, thermal conductance plays an important role in phase stability of 2M WS_2_. We then evaluate the thermal conductivities of 2M WS_2_ flakes on SiO_2_/Si substrates using temperature- and laser-power-dependent Raman spectroscopy^43^. With increase of temperature or laser power, the Raman vibration modes of all WS_2_ flakes with various thicknesses are observed to soften (red shift), as shown in Fig. 4e and Supplementary Fig. 14. We have selected *B*_u_, ${A}_{{{\mbox{g}}}}^{5}$ and ${A}_{{{\mbox{g}}}}^{7}$ modes to plot the peak frequency as functions of temperature or laser power, as shown in Fig. 4f, g and Supplementary Fig. 15a–d. The extracted temperature coefficients (${\chi }_{{{\mbox{T}}}}$) of peak frequencies for different thicknesses are similar, in a range of 0.030–0.060 cm^−1^/K. However, the extracted laser-power coefficients (${\chi }_{{{\mbox{P}}}}$) of peak frequencies exhibit obviously thickness dependence, as shown in Fig. 4f and Supplementary Fig. 15c, d. The ${\chi }_{{{\mbox{P}}}}$ of *B*_u_ mode for 3L, 8L and 22L 2M WS_2_ are 0.585, 1.085, and 1.596 cm^−1^/mW, respectively. Thermal conductance can be estimated by ${\chi }_{{{\mbox{T}}}}$ divided by ${\chi }_{{{\mbox{P}}}}$^33,34,43,44^. Accordingly, the thermal conductance of 3L, 8L, and 22L 2M WS_2_ are calculated to be 0.092, 0.044, and 0.023 mW/K (Fig. 4h), respectively, which clearly evidences thinner sample has higher thermal conductance. To deconvolute thermal conductance of the SiO_2_/Si substrate from our measurements, we also tested Raman spectra of WS_2_ at holey substrates (Supplementary Fig. 16). The as-obtained results also confirm thinner 2M WS_2_ has higher thermal conductance, as shown in Supplementary Fig. 16, except that the calculated thermal conductivities at holey substrates are lower than the corresponding values at conventional SiO_2_/Si substrates. Such reduction of thermal conductance with increase of thickness can be attributed to stronger phonon scattering and enhanced anharmonicity in thicker 2M WS_2_^31^. The high thermal conductance of ultrathin 2M WS_2_ can also be connected with the enhanced intralayer bonding that facilitates intralayer heat conduction. High thermal conductance enables high-efficiency heat dissipation during heating, leading to improved tolerance to elevated temperature for thinner 2M WS_2_.

The different thermal conductivities between thick and thin WS_2_ flakes can also be associated with their distinct phase distributions during the 2M to 2H phase change (Supplementary Fig. 17). Low thermal conductance of a thick 2M WS_2_ flake results in nonuniform heat distribution during continuous heating process, and the 2M to 2H phase change occurs first at regions where heat is concentrated. Moreover, the non-concerted structural evolution among different layers can contribute to high interlayer phonon scattering and cause further low thermal conductance for the intermediate phase WS_2_. Consequently, a sequential phase change at different regions leads to a 2M/2H stripes pattern on the WS_2_ flake (Supplementary Fig. 17). In contrast, high thermal conductance of an ultrathin 2M WS_2_ flake has uniform heat distribution, so the phase change homogeneously occurs at entire region of the flake when phase change temperature is reached (Supplementary Fig. 17).

### Phase transition simulation of different thicknesses WS_2_

To study thickness-dependent phase transition kinetics, we have simulated the 2M to 2H phase change processes for 1L, 2L, and 3L WS_2_ supercells (as shown in supplementary Fig. 18) by employing Vienna ab initio simulation package (VASP) and calculated energies of WS_2_ lattices with different configurations by using climbing image nudged elastic band (CI-NEB) method. Like the transition state theory applied for chemical reactions, these calculations can find the transition state WS_2_ lattice configuration during phase transition and identify the path passing through the transition state and connecting the initial and the final phase states. Such a path consists of a series of phase transition coordinates, including initial phase state (composed by 1T′ layers), final phase state (composed by 1H layers), transition state, and other intermediate lattice configurations. Method and principle used to find these coordinates are discussed in Part III of supporting information with Supplementary Fig. 19. The calculation output electronic files are attached as Supplementary Software 1. Configurations of all WS_2_ lattices at the as-calculated coordinates (coord. I to coord. VII) are shown in Supplementary Figs. 20–26. During the simulated phase transition process, the octahedral coordinated lattices gradually deform until reach the transition states, which further evolve and relax to the trigonal prismatic coordinated lattices. Meanwhile, the WS_2_ unit cell shrinks in *a* and *b* directions (Supplementary Fig. 27), which agrees to the afore-discussed phase transition mechanism.

**Fig. 5:**
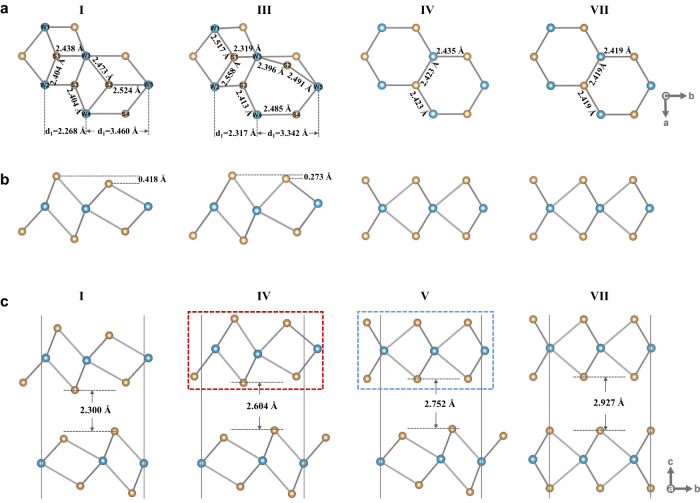
Simulation of 2M to 2H phase transition of WS_2_ using rectangular supercells. Representative molecular geometries of the 1L WS_2_ supercell in the initial 1T′ phase (coord. I), transition state (coord. III), an intermediate configuration (coord. IV) and the final 1H phase (coord. VII), viewing from (**a**) the *c* direction and (**b**) the *a* direction. Process from coord. I to coord. III corresponds to deformation of 1T′-type lattice and process from coord. IV to coord. VII corresponds to relaxation of 1H-type lattice. **c** Molecular geometries of the 2L WS_2_ supercell at the initial phase state (coord. I), transition state (coord. IV), an intermediate configuration (coord. V) and the final phase state (coord. VII), viewing from the *a* direction. The highly deformed layer at the transition state is marked by a red dotted square. The layer turned from 1T′ to 1H type of structures is marked by a blue dotted square. Interlayer spacings are denoted in different states, showing interlayer expansion from coord. I to coord. VII. Lattice edges are depicted in different states, referring to which interlayer dislocation between top and bottom layers can be seen. Color code: blue and orange spheres represent W and S, respectively.

During lattice deformation process of 1L 1T′ WS_2_, W-atom line distance d_1_ increases from 2.280 Å (coord. I) to 2.317 Å (coord. III), while W-atom line distance d_2_ decreases from 3.340 Å (coord. I) to 3.342 Å (coord. III), as shown in Fig. 5a and Supplementary Fig. 20. These results reveal W-atom lines distances tend to be equalized along *b* direction, in consistency with observations from TEM images (Fig. 5a–c) of WS_2_ during 2M to 2H phase transition. With changing of W-atom line distances, S1 and S2 atoms are found to glide along *bc* plane by large ranges from coord. I to III, rendering severe elongation of W2‒S1 bonds to 2.558 Å and breakage of W4‒S2 bonds (Fig. 5a). Meanwhile, the W1‒S1 bond elongates from 2.404 Å (coord. I) to 2.517 Å (coord. III), while W3‒S1 shortens from 2.438 Å (coord. I) to 2.319 Å (coord. III) and W3‒S2 and W5‒S2 bonds also shorten in coord. III. Similarly, W4‒S3 and W4‒S4 bond lengths are found to respectively increase and decrease from coord. I to III. Altering of W‒S bonds lengths also results in out-of-plane displacements of S atoms, which makes distance between different S atomic planes reducing from 0.418 Å at coord. I to 0.273 Å at coord. III (Fig. 5b). The 1L WS_2_ supercell reaches transition state at coord. III, with the highest lattice energy due to the significant lattice deformation. With further atomic gliding and reforming of W‒S bonds, the lattice of 1L WS_2_ turns to 1H type but with stretched W–S bonds in coord. IV (Fig. 5a). After further shortening of W–S bonds in the following three steps (Supplementary Fig. 20), the lattice of 1L WS_2_ finally relaxes to a typical 1H structure in coord. VII (Fig. 5a).

The simulations of 2L and 3L WS_2_ supercells reveal layer-by-layer phase transition mechanism, in good agreement with the XRD and Raman spectra of intermediate phases WS_2_ (Fig. 3g–i). For 2L WS_2_, the top layer changes from the initial 1T′ structure (coord. I) to a deformed structure at coord. IV, where the transition state is reached, and turns to 1H-type structure at coord. V that then relaxes to coord. VII. The bottom layer is gradually distorted from the initial 1T′ structure until coord. IV and changes to 1H structure from coord. VI to VII, as shown in Fig. 5c and Supplementary Figs. 21, 22. Similarly, the bottom layer of 3L WS_2_ first transforms to 1H structure, followed by the mid layer and top layer, seen from Supplementary Figs. 23–26. 1T′/1H heterostructures are seen at coord. V for 2L (Fig. 5c) and 3L WS_2_ (Supplementary Fig. 26). At transition states, both 2L and 3L WS_2_ contain a highly deformed layer, while other layers are also distorted from the initial 1T’ phase, as shown in Supplementary Table 2. The deformed layers in 2L and 3L WS_2_ have larger structural variations from the initial 1T′ lattice compared to that in 1L WS_2_. The two W-atom line distances get closer in transition state 2L and 3L WS_2_ than 1L WS_2_ (Supplementary Table 2). In addition, the deformed layer in 3L WS_2_ have two broken W‒S bonds, while deformed layers in 1L and 2L WS_2_ include only one (Supplementary Table 2).

**Fig. 6:**
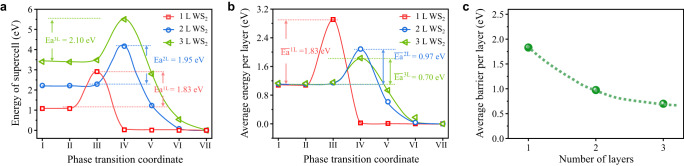
Calculated energy profiles of 2M to 2H phase transition. **a** Total energies and **b** average energies of 1L, 2L, and 3L WS_2_ supercells at the initial state (coord. I), transition states (coord. III for 1L WS_2_ and coord. IV for 2L and 3L WS_2_), final states (coord. VII) and other intermediate configurations. Solid lines are guides to the eye. Energies in (**b**) are normalized by the corresponding numbers of WS_2_ layers. Ea^1L^, Ea^2L^, and Ea^3L^ are total phase transition energy barriers for the 1L, 2L, and 3L WS_2_ supercells, respectively. ${\bar{{{\mbox{Ea}}}}}^{1{{{{{\rm{L}}}}}}}$, ${\bar{{{\mbox{Ea}}}}}^{2{{{{{\rm{L}}}}}}}$ and ${\bar{{{\mbox{Ea}}}}}^{3{{{{{\rm{L}}}}}}}$ are average transition barriers per layer for the 1L, 2L, and 3L WS_2_ supercells, respectively. **c** The average transition barrier per layer as function of the number of layers. A dashed line is a guide to the eye.

The total phase transition barrier of a WS_2_ supercell is defined as the energy difference between the corresponding transition and initial states. As thicker WS_2_ supercell involves larger structural difference between the corresponding transition and initial states, the total phase transition barrier increases from 1.83 eV to 1.95 eV and 2.10 eV, as the number of layers increases from 1L to 2L and 3L, as shown in Fig. 6a. After normalizing the total phase transition barriers by the number of layers, average transition barriers per layer of 1L, 2L, and 3L WS_2_ supercells are calculated to be 1.83 eV, 0.97 eV and 0.70 eV (Fig. 6b), respectively, exhibiting the average transition barrier decreases with the increase of layer number. This result theoretically confirms thinner 2M WS_2_ has higher phase stability. It should be noted that the average transition barrier per layer decreases more and more slowly as the number of layers increases from 1L to 2L and 3L (Fig. 6c), implying the average transition barriers per layer for thick WS_2_ flakes would converge to a certain value. This agrees to the fact that phase transition temperatures of WS_2_ flakes thicker than 5L converge to 120 °C (Fig. 1e).

## Discussion

The higher intralayer bond strength in thinner 2M WS_2_ can be associated with reduction of interlayer vdW interactions. As is known, higher stability of 1T′ TMD than the corresponding 1T TMD originates from the distorted lattice that generates strong intralayer metal–metal bonds and prominent intralayer Coulombic interactions between metal and chalcogen atoms^4^. An interlayer Coulombic interaction, such as vdW force, brings screening effect to the intralayer interactions and weaken the intralayer bond strength^26–28^. As the interlayer vdW interaction decreases with reduction of thickness from bulk to a few layers, the intralayer bond strength accordingly increases and reaches maximum in ML 1T′ TMD, meaning that ML 1T′ TMD has the highest intrinsic phase stability.

VdW interaction also plays an important role in the phase transition process of multilayered WS_2_. One the one hand, vdW-interaction linked layers tend to deform simultaneously during phase transition, resulting in increasing of total transition barrier with the number of layers. One the other hand, the existence of vdW interaction can constrain the freedom of lattice deformation, to minimize the layer stacking energy. Consequently, phase transition of multilayered WS_2_ occurs layer(s) by layer(s). In fact, WS_2_ lattices always tend to minimize the vdW-interaction-associated stacking energy by optimizing the stack configuration. Interlayer expansion and dislocation during phase transition have been demonstrated by the simulated molecular geometries (Fig. 5c and Supplementary Fig. 26) and physical characterizations (Fig. 3d–f and Supplementary Fig. 12) of multilayered WS_2_. For a relatively thick WS_2_ (≥5L), transition state lattice might include two or more highly deformed layers that are widely separated since vdW interactions between them are small. Once the number of deformed layers existing at the transition state turns to be proportionate to the total number of WS_2_ layers, the average phase transition barrier per layer will converge to a constant value for thick and bulk WS_2_ flakes.

In summary, we have demonstrated ultrathin 2M WS_2_ has significantly higher thermal stabilities than the bulk counterparts. Both phase transition temperature and durability of 2M WS_2_ remarkably increase with thickness decreasing from bulk to ML. By analyzing 2M to 2H phase transition mechanism and Raman spectra of 2M WS_2_ with different thicknesses, the higher stability of thinner 2M WS_2_ is associated with stiffened intralayer bonds and enhanced thermal conductance, due to reduction of interlayer interactions. Theoretical calculation indicates average transition barrier increases with reduction of WS_2_ layer number, since the phase transition occurs through a layer(s)-by-layer(s) mechanism. The high intrinsic phase stabilities of ultrathin 2M TMDs can inspire their tempting applications in various fields, including superconductor, electronics and energy conversion and storage.

## Methods

### Fabrication of different layered 2M WS_2_

K_0.7_WS_2_ crystals were synthesized as previously reported^7^. Specifically, K_2_S_2_ (prepared via liquid ammonia), W (99.9%, Alfa Aesar), and S (99.99%, Alfa Aesar) were mixed by the stoichiometric ratios and ground in an argon-filled glovebox. The mixtures were pressed into a pellet and sealed in the evacuated quartz tube. The tube was heated at 850 °C for 2000 min and slowly cooled to 550 °C at a rate of 0.1 °C min^−1^. 2M WS_2_ single crystals were obtained by oxidizing K_0.7_WS_2_ in an aqueous solution containing 0.01 M K_2_Cr_2_O_7_ and 0.02M H_2_SO_4_ at room temperature for 1 h. Different layered WS_2_ flakes were mechanically exfoliated from the synthesized bulk 2M WS_2_ onto a SiO_2_/Si substrate or a holey SiO_2_/Si substrate. To transfer the exfoliated WS_2_ from SiO_2_/Si to PDMS, the SiO_2_/Si substrate was spin-coated with PMMA film and etched by KOH solution. A piece of PDMS film was used to pick up the WS_2_/PMMA and the PMMA film was removed by soaking in acetone for an hour. More details about sample preparation are available in Supplementary Note 1.

### Temperature- and laser-power-dependent Raman measurements

Micro-Raman spectra were taken on the 2M WS_2_ using a confocal microscope Raman system (WITec, Alpha300R) with an optical microscope (Nikon). For temperature-dependent Raman measurement, the exfoliated 2M WS_2_ on a Si/SiO_2_ or holey Si/SiO_2_ or PDMS substrate was put on a hot plate, and temperature was increased by 5 °C and held for 1 min or 15 min in each heating step. The heating treatment was carried out either in the air or in a glovebox with an Ar atmosphere. After heating, the sample was transferred to the Raman instrument for Raman spectra acquisition. For laser-power-dependent Raman measurement, the powers of laser irradiating on the WS_2_ flakes were quantified by a power meter (Thorlabs). More details about Raman measurements are available in Supplementary Note 2.

### Physical characterizations

The morphology and microstructure of the 2M WS_2_ flakes were transferred to microgrid and examined by transmission electron microscopy (TEM, FEI Verios G4). The thickness and morphology were analyzed by using AFM images, which were captured with a Bruker Dimension Icon AFM under tapping mode. The samples were characterized by XRD pattern (Bruker D8 Advanced).

### Computational method

All the calculations were performed by the employee of the VASP^45^. The Perdew–Burke–Ernzerhof form of the generalized gradient approximation was employed to describe electronic exchange and correlation^46^. Lattice constants and atom positions of WS_2_ in 2H and 2M phases, respectively, were optimized by using the conjugate gradient algorithm until the maximum force on a single atom is less than 0.02 eV/Å. The thickness of the vacuum layer exceeded 15 Å to eliminate the fictitious interaction caused by periodic cells. The van der Waals interactions were depicted in terms of the D_3_ method of Grimme^47^. The CI-NEB method was adopted to locate the transition state from 2H to 2M^48^. More details about computational methods are available in Supplementary Note 3.

### Supplementary information

Supplementary Information

Peer Review File

Description of Additional Supplementary Files

Supplementary Software 1

## Data Availability

All data supporting the findings of this study are available within the paper and the supplementary information files. Additional data are available from the corresponding authors upon request.

## References

[1] Voiry D, Mohite A, Chhowalla M (2015). Phase engineering of transition metal dichalcogenides. Chem. Soc. Rev..

[2] Yang H, Kim SW, Chhowalla M, Lee YH (2017). Structural and quantum-state phase transitions in van der Waals layered materials. Nat. Phys..

[3] Lai Z (2021). Metastable 1T’-phase group VIB transition metal dichalcogenide crystals. Nat. Mater..

[4] Zhao W (2018). Metastable MoS_2_: crystal structure, electronic band structure, synthetic approach and intriguing physical properties. Chem. Eur J.

[5] Liu L (2018). Phase-selective synthesis of 1T’ MoS_2_ monolayers and heterophase bilayers. Nat. Mater..

[6] Yu Y (2018). High phase-purity 1T’-MoS_2_- and 1T’-MoSe_2_- layered crystals. Nat. Chem..

[7] Fang Y (2019). Discovery of superconductivity in 2M WS_2_ with possible topological surface states. Adv. Mater..

[8] Shang C (2018). Superconductivity in the metastable 1T’ and 1T”‘ phases of MoS_2_ crystals. Phys. Rev. B.

[9] Li YW (2021). Observation of topological superconductivity in a stoichiometric transition metal dichalcogenide 2M-WS_2_. Nat. Commun..

[10] Yuan Y (2019). Evidence of anisotropic Majorana bound states in 2M-WS_2_. Nat. Phys..

[11] Soluyanov AA (2015). Type-II Weyl semimetals. Nature.

[12] Sun Y, Wu S-C, Ali MN, Felser C, Yan B (2015). Prediction of Weyl semimetal in orthorhombic MoTe_2_. Phys. Rev. B.

[13] Tan SJR (2017). Chemical stabilization of 1T’ phase transition metal dichalcogenides with giant optical Kerr nonlinearity. J. Am. Chem. Soc..

[14] Fang Y (2019). Structural determination and nonlinear optical properties of new 1T “‘-type MoS_2_ compound. J. Am. Chem. Soc..

[15] Liu Z (2022). General bottom-up colloidal synthesis of nano-monolayer transition-metal dichalcogenides with high 1T’-phase purity. J. Am. Chem. Soc..

[16] Chou SS (2015). Understanding catalysis in a multiphasic two-dimensional transition metal dichalcogenide. Nat. Commun..

[17] Acerce M, Voiry D, Chhowalla M (2015). Metallic 1T phase MoS_2_ nanosheets as supercapacitor electrode materials. Nat. Nanotechnol..

[18] Chen W (2022). Two-dimensional quantum-sheet films with sub-1.2 nm channels for ultrahigh-rate electrochemical capacitance. Nat. Nanotechnol..

[19] Luo H, Yu P, Li G, Yan K (2022). Topological quantum materials for energy conversion and storage. Nat. Rev. Phys..

[20] Lin Y-C, Dumcencon DO, Huang Y-S, Suenaga K (2014). Atomic mechanism of the semiconducting-to-metallic phase transition in single-layered MoS_2_. Nat. Nanotechnol..

[21] Kappera R (2014). Phase-engineered low-resistance contacts for ultrathin MoS_2_ transistors. Nat. Mater..

[22] Apte A (2018). Structural phase transformation in strained monolayer MoWSe_2_ alloy. ACS Nano.

[23] Song S (2016). Room temperature semiconductor-metal transition of MoTe_2_ thin films engineered by strain. Nano Lett..

[24] Lin Y-C (2018). Stable 1T tungsten disulfide monolayer and its junctions: growth and atomic structures. Acs Nano.

[25] Yang J (2022). Phase engineering of metastable transition metal dichalcogenides via ionic liquid assisted synthesis. ACS Nano.

[26] Cheiwchanchamnangij T, Lambrecht WRL (2012). Quasiparticle band structure calculation of monolayer, bilayer, and bulk MoS_2_. Phys. Rev. B.

[27] Mak KF (2013). Tightly bound trions in monolayer MoS_2_. Nat. Mater..

[28] Lee C (2010). Anomalous lattice vibrations of single- and few-layer MoS_2_. ACS Nano.

[29] He Y, He Q, Wang L, Zhu C, Liu Z (2019). Self-gating in semiconductor electrocatalysis. Nat. Mater..

[30] Liu X (2019). The critical role of electrolyte gating on the hydrogen evolution performance of monolayer MoS_2_. Nano Letters.

[31] Gu X, Li B, Yang R (2016). Layer thickness-dependent phonon properties and thermal conductivity of MoS_2_. J. Appl. Phys..

[32] Wu C, Liu C, Tao Y, Zhang Y, Chen Y (2020). Anomalous layer thickness-dependent thermal conductivity of Td-WTe_2_ through first-principles calculation. Phys. Lett. A.

[33] Zhou S, Tao X, Gu Y (2016). Thickness-dependent thermal conductivity of suspended two-dimensional single-crystal In_2_Se_3_ layers grown by chemical Vapor deposition. J. Phys. Chem. C.

[34] Zou B (2020). Thickness-dependent ultralow in-plane thermal conductivity of chemical vapor-deposited SnSe_2_ nanofilms: implications for thermoelectrics. ACS Appl. Nano Mater..

[35] Xiong F (2015). Li Intercalation in MoS_2_: in situ observation of its dynamics and tuning optical and electrical properties. Nano Lett..

[36] Gutierrez HR (2013). Extraordinary room-temperature photoluminescence in triangular WS_2_ monolayers. Nano Lett..

[37] Pierucci D (2019). Evidence for a narrow band gap phase in 1T’ WS2 nanosheet. Appl. Phys. Lett..

[38] Song X (2023). Synthesis of an aqueous, air-stable, superconducting 1T’-WS_2_ monolayer ink. Sci. Adv..

[39] Friedman AL (2017). Evidence for chemical vapor-induced 2H to 1T phase transition in MoX_2_ (X = Se, S) transition metal dichalcogenide films. Sci. Rep..

[40] Nurdiwijayanto L, Ma R, Sakai N, Sasaki T (2018). Insight into the structural and electronic nature of chemically exfoliated molybdenum disulfide nanosheets in aqueous dispersions. Dalton Trans..

[41] Shi S, Sun Z, Hu YH (2018). Synthesis, stabilization and applications of 2-dimensional 1T metallic MoS_2_. J. Mater. Chem. A.

[42] Schutte WJ, Deboer JL, Jellinek F (1987). Crystal structures of tungsten disulfide and diselenide. J. Solid State Chem..

[43] Yan R (2014). Thermal conductivity of monolayer molybdenum disulfide obtained from temperature-dependent Raman spectroscopy. ACS Nano.

[44] Bae JJ (2017). Thickness-dependent in-plane thermal conductivity of suspended MoS_2_ grown by chemical vapor deposition. Nanoscale.

[45] Kresse G, Furthmuller J (1996). Efficient iterative schemes for ab initio total-energy calculations using a plane-wave basis set. Phys. Rev. B.

[46] Perdew JP, Burke K, Ernzerhof M (1996). Generalized gradient approximation made simple. Phys. Rev. Lett..

[47] Grimme S (2006). Semiempirical GGA-type density functional constructed with a long-range dispersion correction. J. Comput. Chem..

[48] Henkelman G, Uberuaga BP, Jonsson H (2000). A climbing image nudged elastic band method for finding saddle points and minimum energy paths. J. Chem. Phys..

