# Supplementary material for: High intrinsic phase stability of ultrathin 2M WS2
Source: Nat Commun. 2024 Feb 10;15:1263. doi: 10.1038/s41467-024-45676-3 (PMC10858873; doi:10.1038/s41467-024-45676-3)
Supplement: Supplementary file 1 — Supplementary Information [file 41467_2024_45676_MOESM1_ESM.pdf]

Supplementary Information:

High Intrinsic Phase Stability of Ultrathin 2M WS_2

Xiangye Liu[1, 2]†, Pingting Zhang[1, 2]†, Shiyao Wang[3]†, Yuqiang Fang[4, 5]†, Penghui Wu[1, 2], Yue Xiang[1, 2], Jipeng Chen[1, 2], Chendong Zhao[4], Xian Zhang[5], Wei Zhao[4], Junjie Wang[3], Fuqiang Huang[4], Cao Guan[1, 2]*

[1]Institute of Flexible Electronics, Northwestern Polytechnical University, Xi'an 710072, China.

[2]Key laboratory of Flexible Electronics of Zhejiang Province, Ningbo Institute of Northwestern Polytechnical University, 218 Qingyi Road, Ningbo, 315103, China.

[3]State Key Laboratory of Solidification Processing, Northwestern Polytechnical University, Xi'an, Shaanxi 710072, China.

[4]State Key Laboratory of High-Performance Ceramics and Superfine Microstructure, Shanghai Institute of Ceramics, Chinese Academy of Sciences Shanghai 200050, China.

[5]Qian Xuesen Laboratory of Space Technology, China Academy of Space Technology, Beijing 100094, China.

[6]These authors contribute equally: Xiangye Liu, Pingting Zhang, Shiyao Wang and Yuqiang Fang.

Corresponding Author

*E-mail: iamcguan@nwpu.edu.cn (C. Guan)

Content

Supplementary Note 1. Fabrication of different layered 2M WS_2 3

1.1 Synthesis of high purity 2M WS_2 single crystal 3

1.2 Mechanical exfoliation and transfer of 2M WS_2 3

Supplementary Note 2. Temperature- and laser-power- dependent Raman and durability measurements 4

Supplementary Note 3. Computational method 5

IV. Supplementary figures 7

Supplementary Note 1. Fabrication of different layered 2M WS_2

1.1 Synthesis of high purity 2M WS_2 single crystal

As we previously reported[1], 2M WS_2 single crystals were prepared by deintercalation of interlayer potassium cations from $K_{0.7}WS_2$ crystals. For the synthesis of $K_{0.7}WS_2$, K_2S_2 (prepared via liquid ammonia), W (99.9%, Macklin) and S (99.99%, Macklin) were mixed by the stoichiometric ratios and ground in an argon-filled glovebox. The mixtures were pressed into a pellet and sealed in the evacuated quartz tube. The tube was heated at 850 °C for 2000 min and slowly cooled to 550 °C at a rate of 0.1 °C min^{-1}. The synthesized $K_{0.7}WS_2$ (0.1 g) was oxidized chemically by $K_2Cr_2O_7$ (0.01 mol L^{-1}) in aqueous H_2SO_4 (50 mL, 0.02 mol L^{-1}) at room temperature for 1 h. Finally, the 2M WS_2 crystals were obtained after washing in distilled water several times and drying in the vacuum oven at room temperature.

1.2 Mechanical exfoliation and transfer of 2M WS_2

Different layered WS_2 flakes were mechanically exfoliated from the synthesized bulk WS_2 onto a Si substrate covered with 300 nm SiO_2, or on a holey SiO_2/Si substrate. In a typical run, pieces of bulk WS_2 were put on the sticky side of a piece of adhesive tape, followed by repeated folding and unfolding of the tape to produce thin flakes along the tape's surface. The tape was then pressed onto the substrate. After heating at 50 °C for 5 minutes, the tape was removed from the substrate, leaving thin layered WS_2 flakes on the substrate.

To transfer the exfoliated WS_2 from SiO_2/Si to polydimethylsiloxane (PDMS), the SiO_2/Si substrate was spin-coated with PMMA film and annealed at 80 ° C for 5 min. The PMMA precursor used in this experiment is 950 K A4, and the spin-coating was performed at 2000 rpm for 1 min. After etching with KOH solution, WS_2/PMMA film was floating on surface of the

solution. A piece of PDMS film was used to pick up the WS_2/PMMA and it was further annealed at 80 °C for 5 min. Finally, the sample was soaked in acetone for an hour to remove the PMMA film.

Supplementary Note 2. Temperature- and laser-power- dependent Raman and durability measurements

Micro-Raman spectra were taken on the 2M WS_2 using a confocal microscope Raman system (WITec, Alpha300R) with optical microscope (Nikon). The measurements were performed with laser excitation wavelength of 532 nm and 100× objective lens (NA = 0.9). The output power of laser is set to 1 mW for acquisitions of all spectra except for the laser-power-dependent Raman measurements. The Raman signals from the sample were introduced to an electron multiplying charge coupled device (CCD) detector (Andor) through a grating with 600 grooves per millimiter. The CCD integration was set to two times with exposure for 10 s in each time for acquisitions of all spectra. The Si peak at 520 cm^{-1} was used as a reference for calibration of wavenumber.

For temperature-dependent Raman measurements, the exfoliated 2M WS_2 on a Si/SiO_2 or holey Si/SiO_2 or PDMS substrate was put on a hot plate and temperature was increased by 5 °C and held for 1 min or 15 min in each heating step. The heating treatment was carried out either in the air or in a glovebox with argon (Ar) atmosphere. After heating, the sample was transferred to the Raman instrument for Raman spectra acquisition.

For laser-power-dependent Raman measurements, the exfoliated 2M WS_2 flakes on the Si/SiO_2 or holey Si/SiO_2 or PDMS substrates were measured by setting the laser at specific output powers. The exact laser power exposed by a WS_2 sample was measured by a power meter

(Thorlabs), and all the data obtained from laser-power-dependent Raman measurements were based on the actual laser intensity irradiating on the WS_2 samples.

Supplementary Note 3. Computational method

Theoretical simulation of 2M to 2H phase transition of WS_2 is based on the transition state theory[2]. The initial 2M state can reach the final 2H state through many different pathways on the potential energy surface (PES), as shown in Supplementary Fig. 18. On any pathway connecting initial and final states on the PES, there will be a state corresponding to the energy maximum (EM). The transition state has an energy equal to the lowest EM. Therefore, the transition state can be located by finding the lowest EM on the connection pathways as shown in Supplementary Fig. 18. To locate the transition state from 2H to 2M, the climbing image nudged elastic band (CI-NEB) method was adopted[3]. Five images were used to build the elastic band between the given initial and final states along phase transition coordinates path. The energy and force convergence criteria were set to 10^{-5} eV and 0.05 eV/Å, respectively.

All the calculations were performed by the employ of the Vienna ab initio simulation package (VASP)[4]. The projector augmented wave pseudopotentials were used to describe the interactions between valence electrons and ionic cores[5]. The Perdew-Burke-Ernzerhof form of the generalized gradient approximation (GGA) was employed to describe electronic exchange and correlation[6]. The cutoff energy for the plane-wave basis was set to be 520 eV. The Γ-centered $10 \times 6 \times 1$ k-point mesh was used to sample the Brillouin zone (BZ) by employing the Monkhorst-Pack method[7]. All the lattice constants and atom positions of different thicknessed WS_2 in 2H and 2M phases respectively were optimized by using the conjugate gradient algorithm. The convergence criteria for energy and atom forces were 10^{-5} eV and 0.02 eV/Å, respectively. The thickness of the vacuum layer exceeded 15 Å to eliminate the fictitious interaction caused by

periodic cells. The Brillouin-zone integrations were approximated by Γ-centered k-point sampling of the Monkhorst-Pack scheme with a k-point mesh resolution of $2\pi \times 0.03$ Å^{-1}.[7] The van der Waals interactions were depicted in terms of the D_3 method of Grimme[8].

IV. Supplementary figures

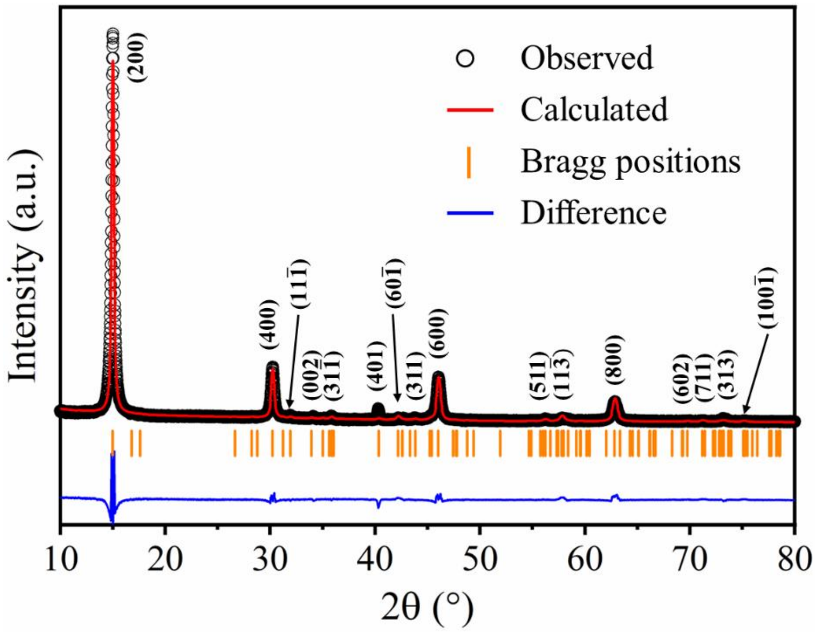

Supplementary Fig. 1. Rietveld refinement of powder XRD pattern in $C_{2/m}$ space group of the synthesized 2M WS_2.

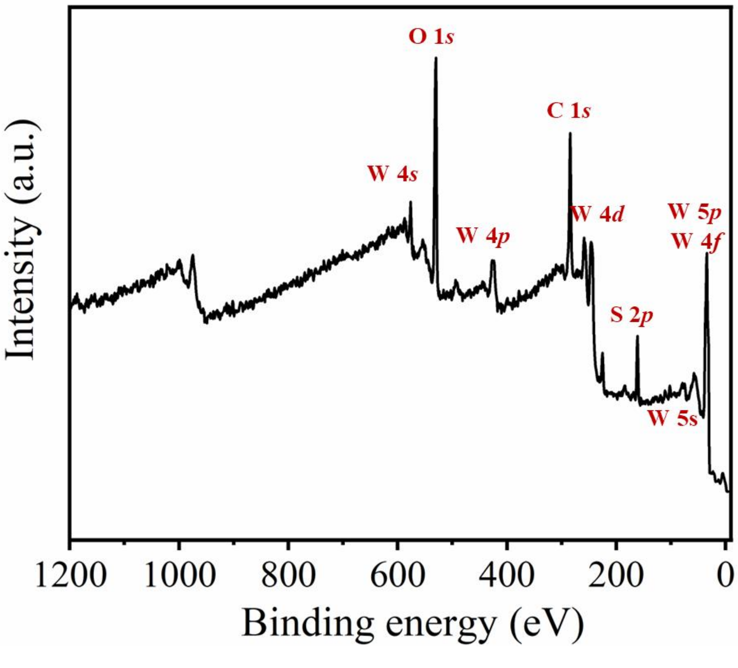

Supplementary Fig. 2. XPS survey of the synthesized 2M WS_2.

Supplementary Table 1. Structural parameters obtained from Rietveld refinements of the powder XRD of 2M WS_2.

R values	R_p=10.16	R_{wp}	R_{exp}	
	10.16	13.03	7.27	
Lattice parameters	*a* (Å)	*b* (Å)	*c* (Å)	*β* (°)
	12.8471	3.2177	5.6912	112.8368
Atom sites	*x*	*y*	*z*	
W	0.75566	0.50000	0.79548	
S1	0.13880	0.00000	0.32374	
S2	0.39484	0.00000	0.21906	

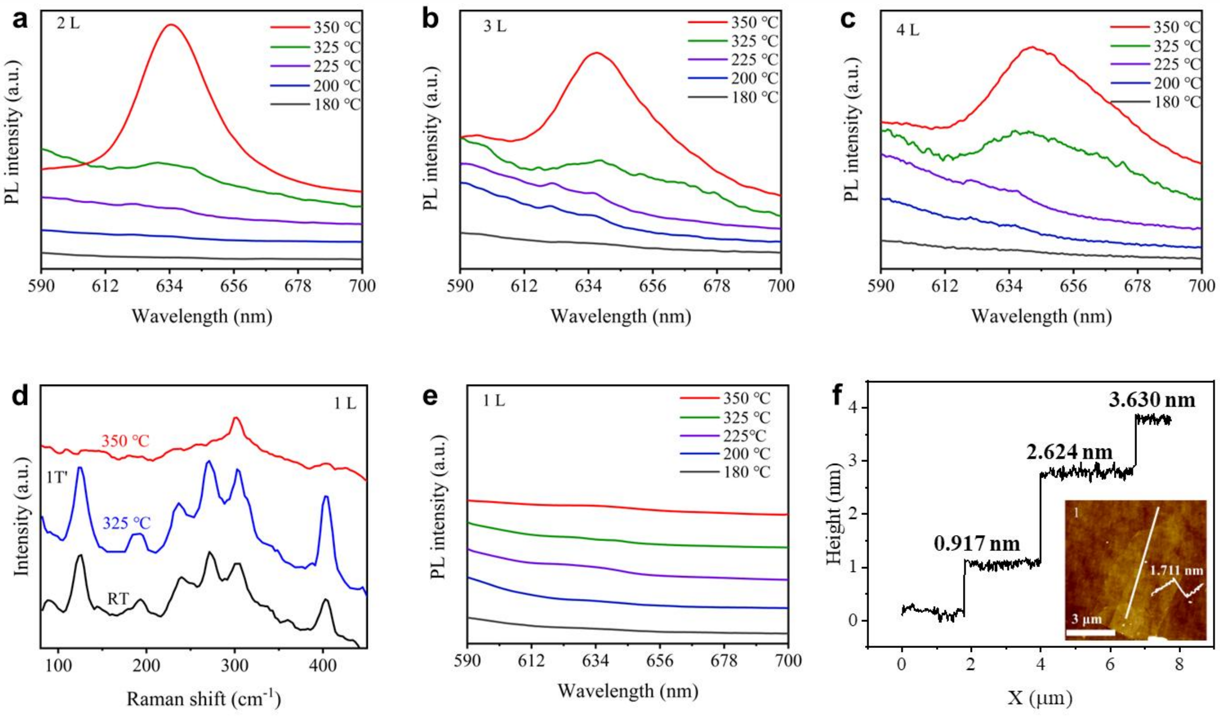

Supplementary Fig. 3. PL spectra of (a) 2 L (b) 3 L (c) 4 L 2M WS_2 in Fig. 1a heated at different temperatures in the air. (d) Raman and (e) PL spectra of ML 1T′ WS_2 in Fig. 1a heated at different temperatures in the air, evidencing the ML 1T′ WS_2 is stable before 350 °C in the air. (f) Height profiles of the AFM image shown in Fig. 1b.

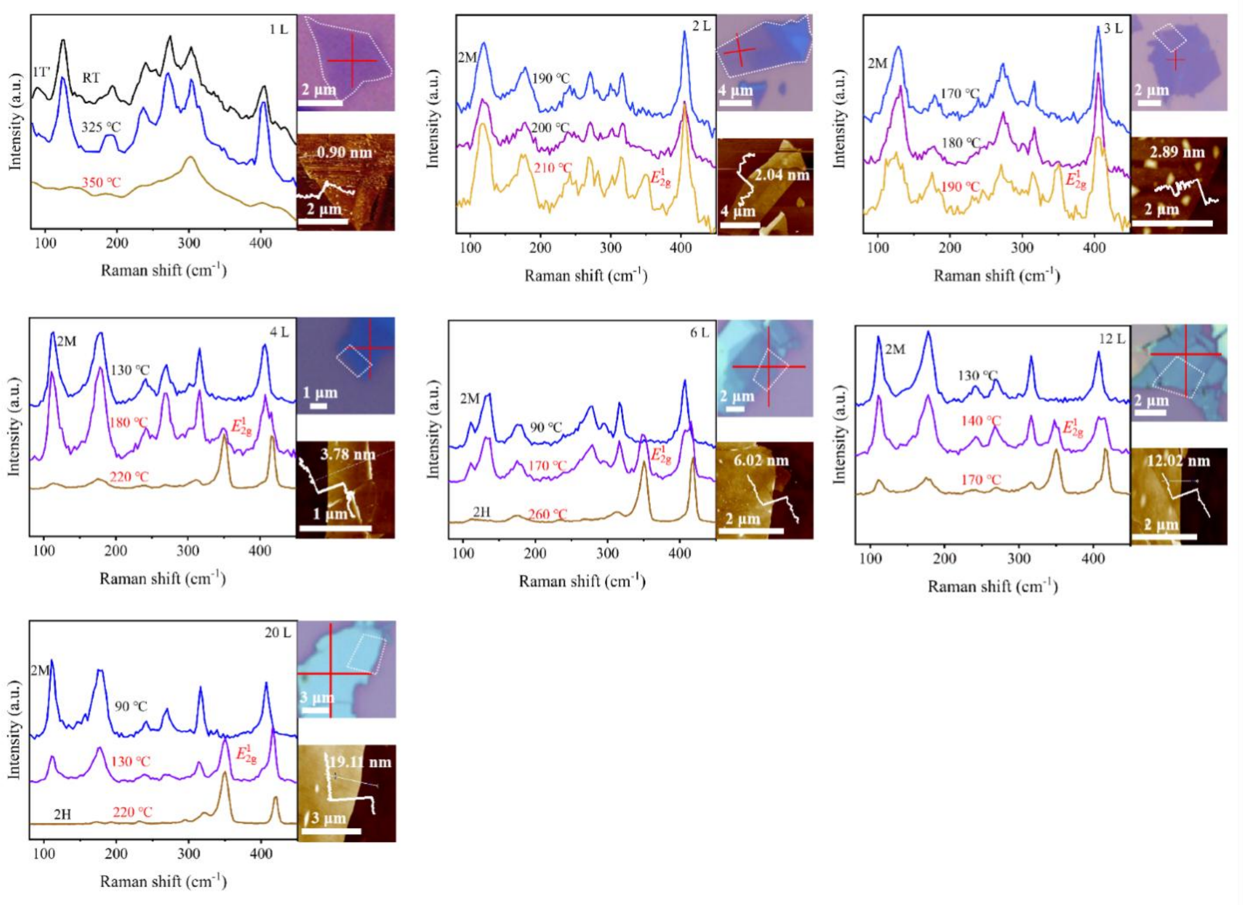

Supplementary Fig. 4. Optical and AFM images and the corresponding height profiles at room temperature and Raman spectra of 2M (or 1T′) WS_2 flakes with various thicknesses on Si/SiO_2 substrates after heated at different temperatures for 1 min in the air. AFM measured regions are labeled with dotted squares in the corresponding optical images. Activation of 2M to 2H phase transition is indicated by emergence of the E_{2g}^{1} mode at 350.2 cm^{-1}. Various intermediate phases WS_2 are formed by heating, and higher intensity of E_{2g}^{1} mode means larger extent of 2M to 2H phase transition.

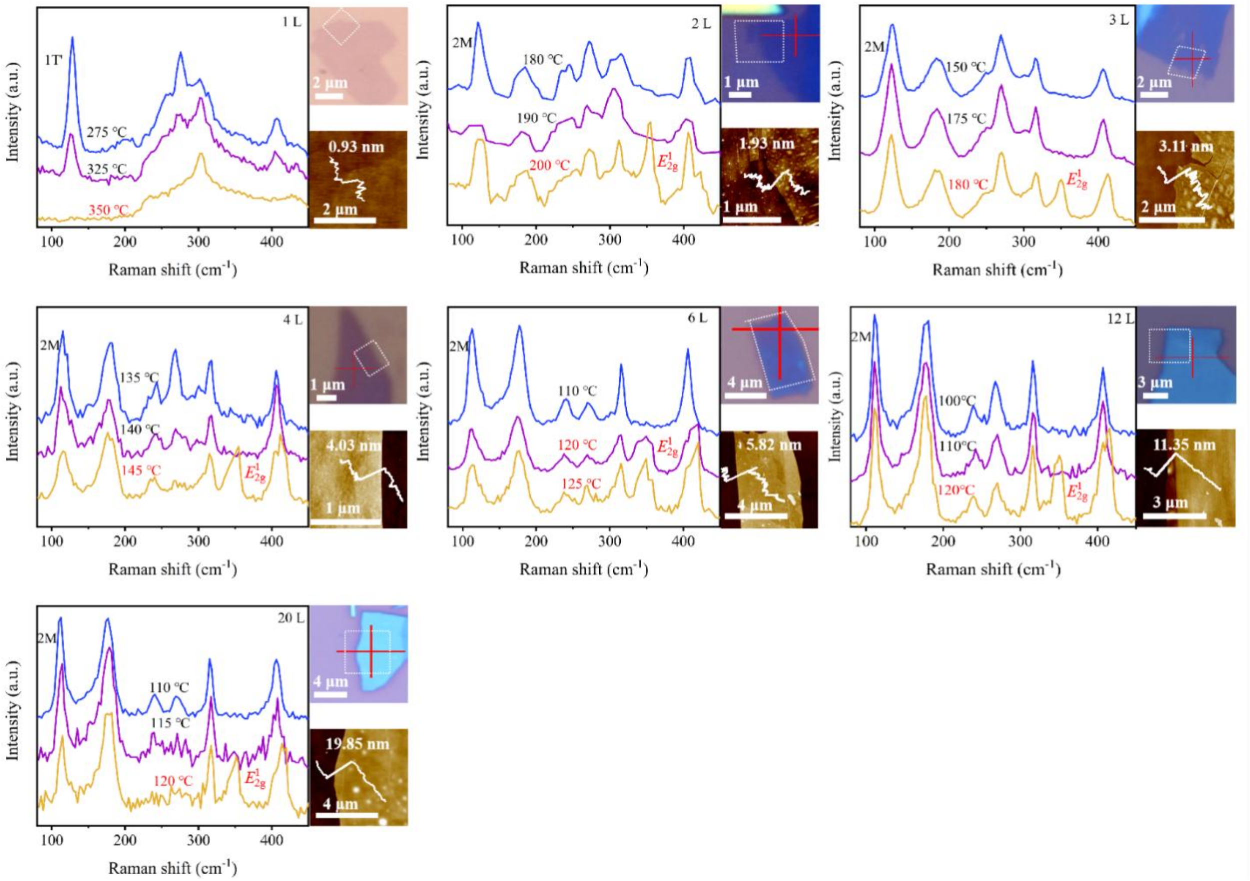

Supplementary Fig. 5. Optical and AFM images and the corresponding height profiles at room temperature and Raman spectra of 2M (or 1T′) WS_2 flakes with various thicknesses on Si/SiO_2 substrates after heated at different temperatures for 15 min in the air. AFM measured regions are labeled with dotted squares in the corresponding optical images. Activation of 2M to 2H phase transition is indicated by emergence of the E^1_{2g} mode at 350.2 cm^{-1}. Various intermediate phases WS_2 are formed by heating, and higher intensity of E^1_{2g} mode means larger extent of 2M to 2H phase transition.

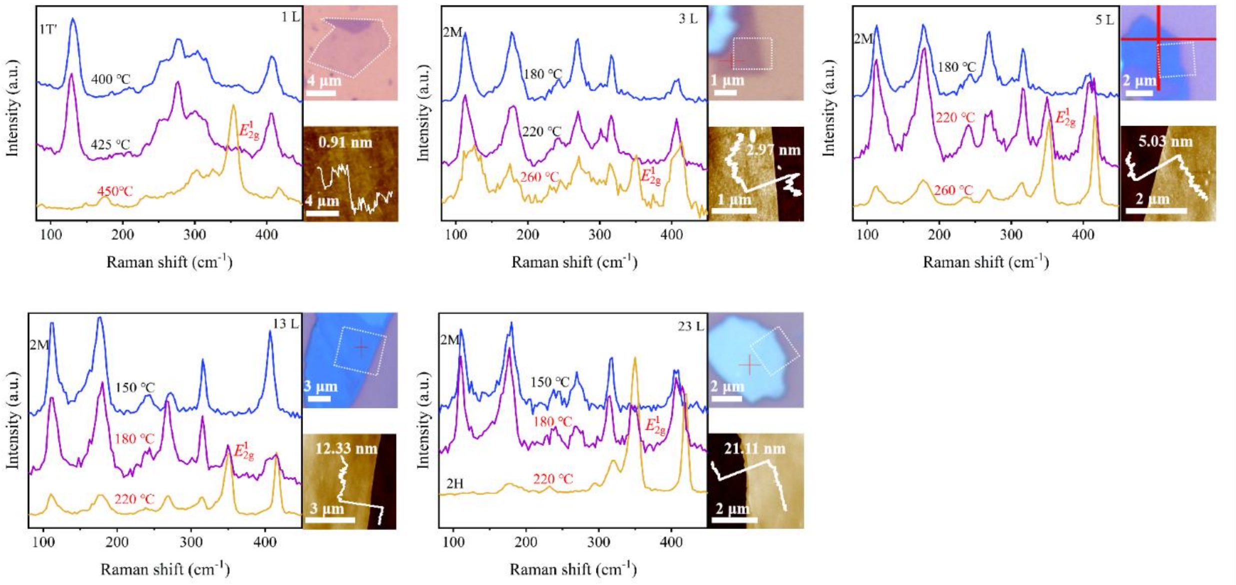

Supplementary Fig. 6. Optical and AFM images and the corresponding height profiles at room temperature and Raman spectra of 2M (or 1T′) WS_2 flakes with various thicknesses on Si/SiO_2 substrates after heated at different temperatures for 1 min in Ar atmosphere. AFM measured regions are labeled with dotted squares in the corresponding optical images. Activation of 2M to 2H phase transition is indicated by emergence of the E_{2g}^{1} mode at 350.2 cm^{-1}. Various intermediate phases WS_2 are formed by heating, and higher intensity of E_{2g}^{1} mode means larger extent of 2M to 2H phase transition.

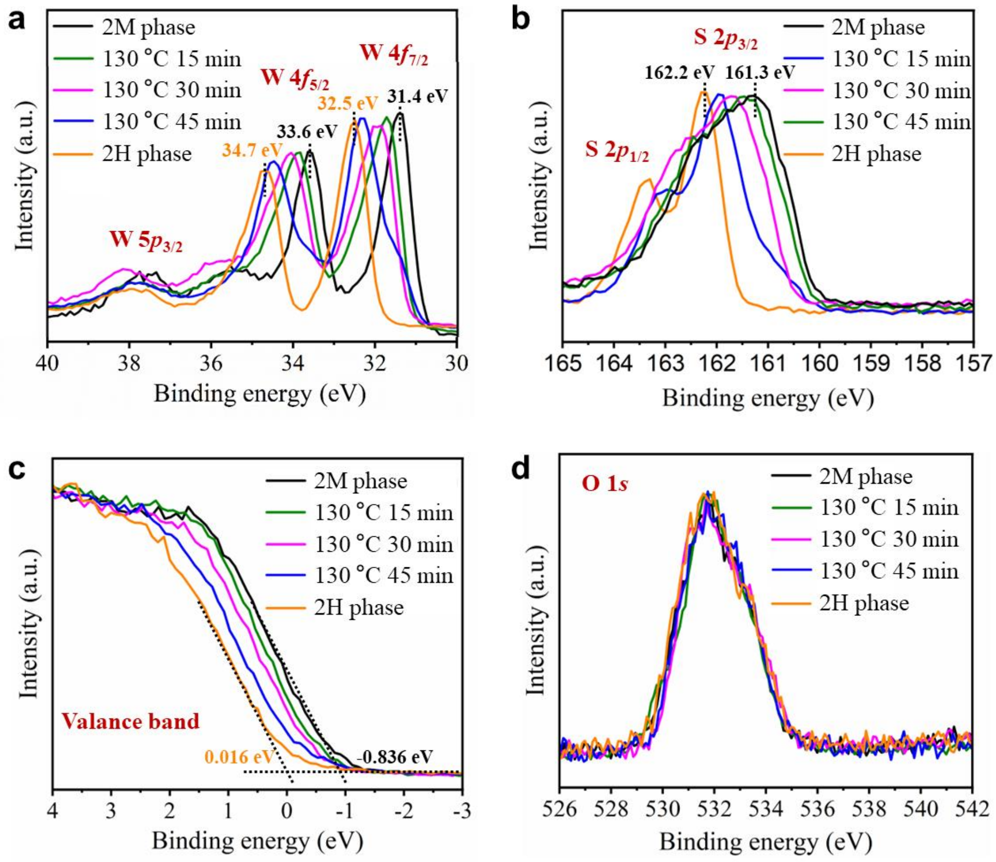

Supplementary Fig. 7. High-resolution XPS spectra of (a) W 4f-5p, (b) S 2p, (c) valance band and (d) O 1s of 2M, intermediate and 2H phases bulk WS_2. Intermediate phases WS_2 were obtained by heating 2M WS_2 in the air at 130 °C for 15 min, 30 min, and 45 min, respectively. 2H phase WS2 were obtained by heating 2M WS_2 in the air at 250 °C for 20 min.

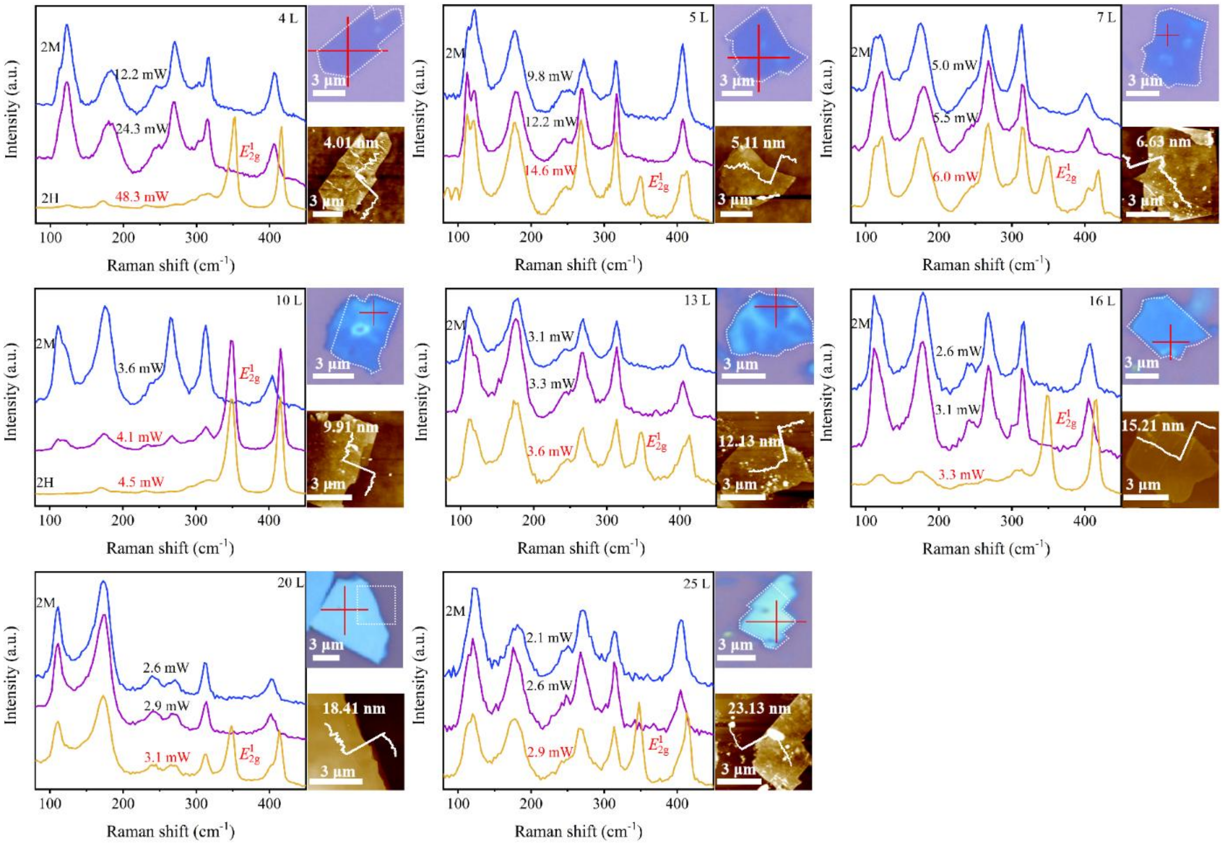

Supplementary Fig. 8. Optical and AFM images and the corresponding height profiles at room temperature and Raman spectra of 2M WS_2 flakes with various thicknesses on Si/SiO_2 substrates, measured with different powers of incident laser. AFM measured regions are labeled with dotted squares in the corresponding optical images. Activation of 2M to 2H phase transition is indicated by emergence of the E^1_{2g} mode at 350.2 cm^{-1}. Various intermediate phases WS_2 are formed by heating, and higher intensity of E^1_{2g} mode means larger extent of 2M to 2H phase transition.

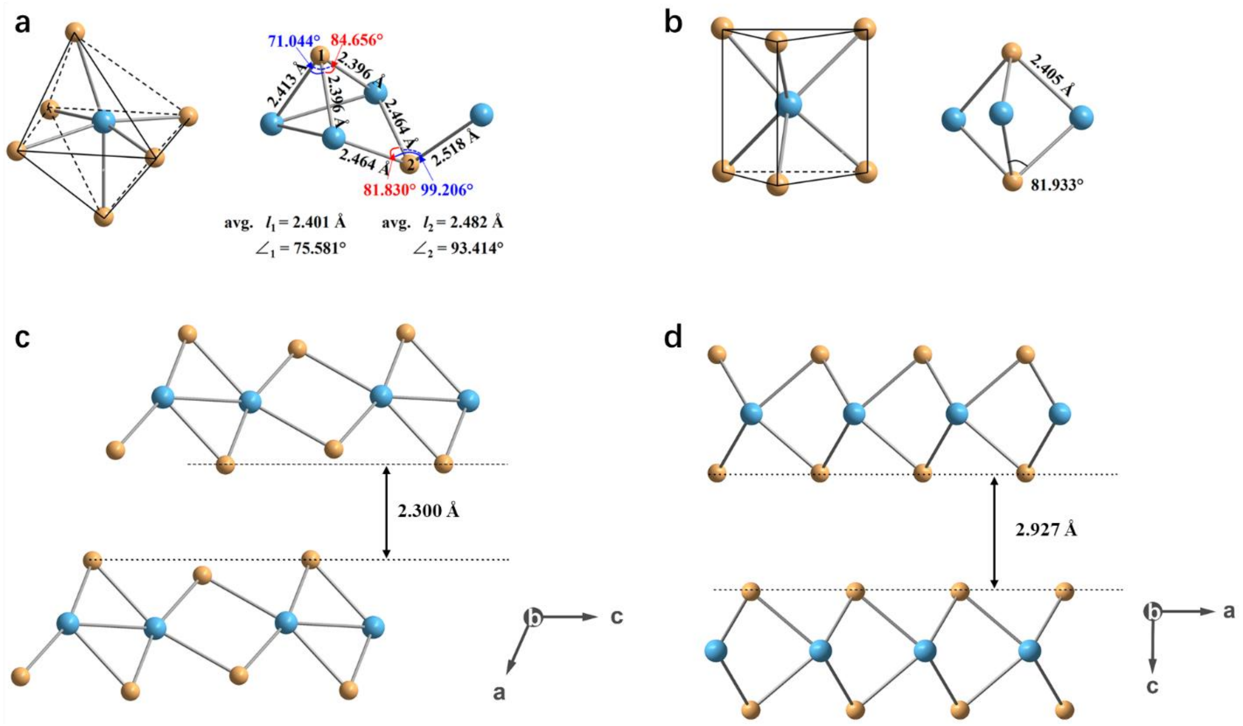

Supplementary Fig. 9. (a) Distorted octahedral coordinated W and S atoms and representative W–S bond lengths and W–S–W angles and the average values and (c) the interlayer spacing of 2M WS_2. S1 atoms locate in C or A' planes and S2 atoms locate in C' or A planes as defined in Fig. 2a. (b) Trigonal prismatic coordinated W and S atoms and W–S bond length and W–S–W angle and (d) the interlayer spacing of 2H WS_2.[9] Color code: blue and orange spheres represent W and S, respectively.

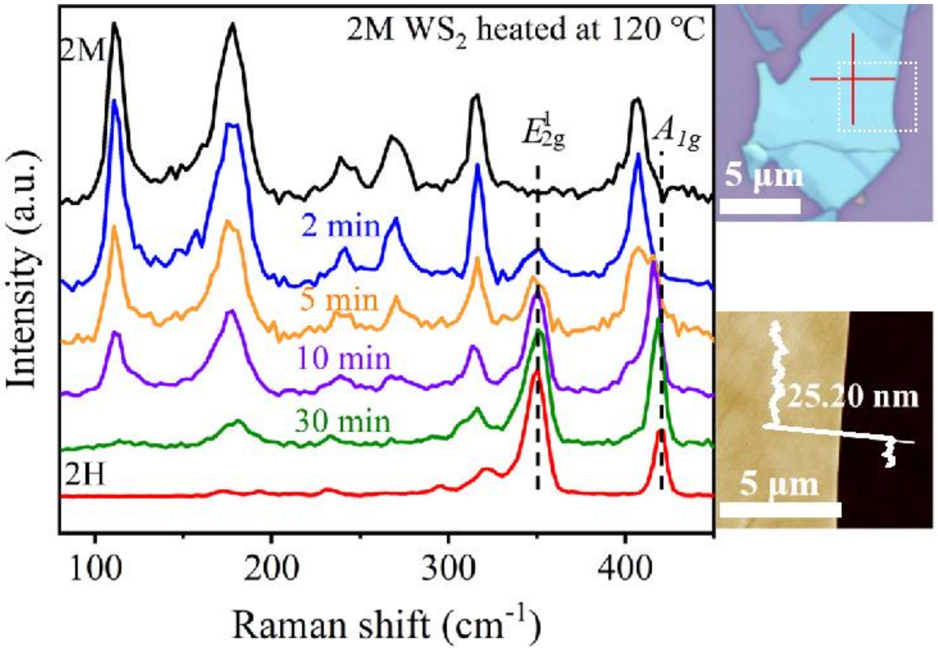

Supplementary Fig. 10. Optical and AFM images with a height profile at room temperature and Raman spectra of a piece of multilayered WS_2 flake in 2M, intermediate and 2H phases. 2H and intermediate phases WS_2 were obtained by heating the 2M WS_2 in the air at 250 °C for 20 min and at 120 °C for various times, respectively. AFM measured regions are labeled with dotted squares in the corresponding optical images. A_{1g} mode exhibits blue shifts as the extent of 2M to 2H phase change increases.

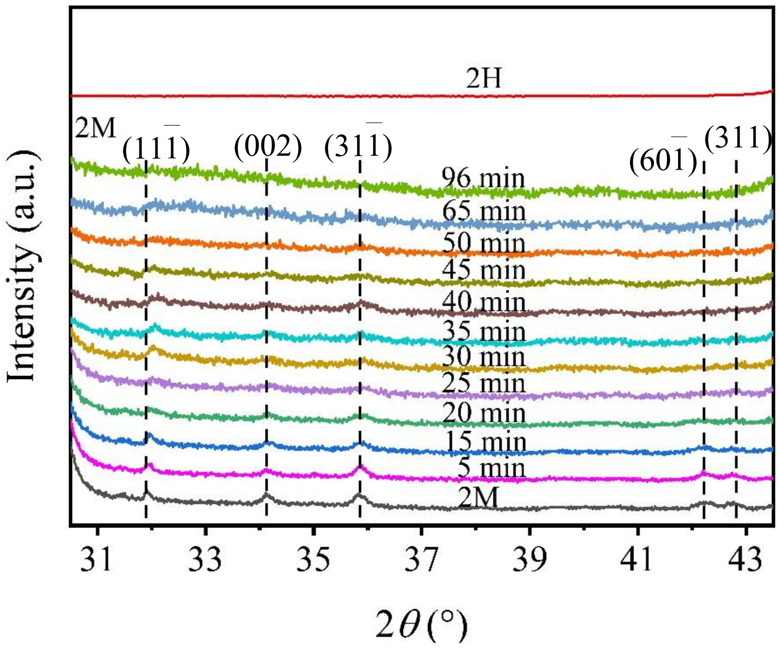

Supplementary Fig. 11. A zoomed-in view of Fig. 3g in the range of 31° ~ 43°.

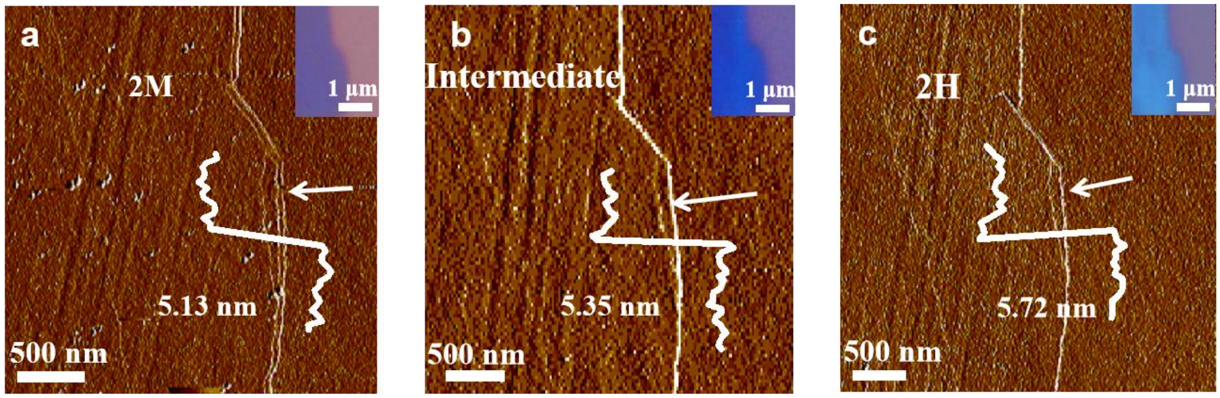

Supplementary Fig. 12. AFM images with height profiles and optical images (insets at top-right corners) of a piece of a 6 L WS_2 flake in (a) 2M, (b) an intermediate and (c) 2H phases. Thicknesses of this flake at different phases are labeled. Edge of the WS_2 flake is denoted by white arrow, where interlayer sliding can be seen.

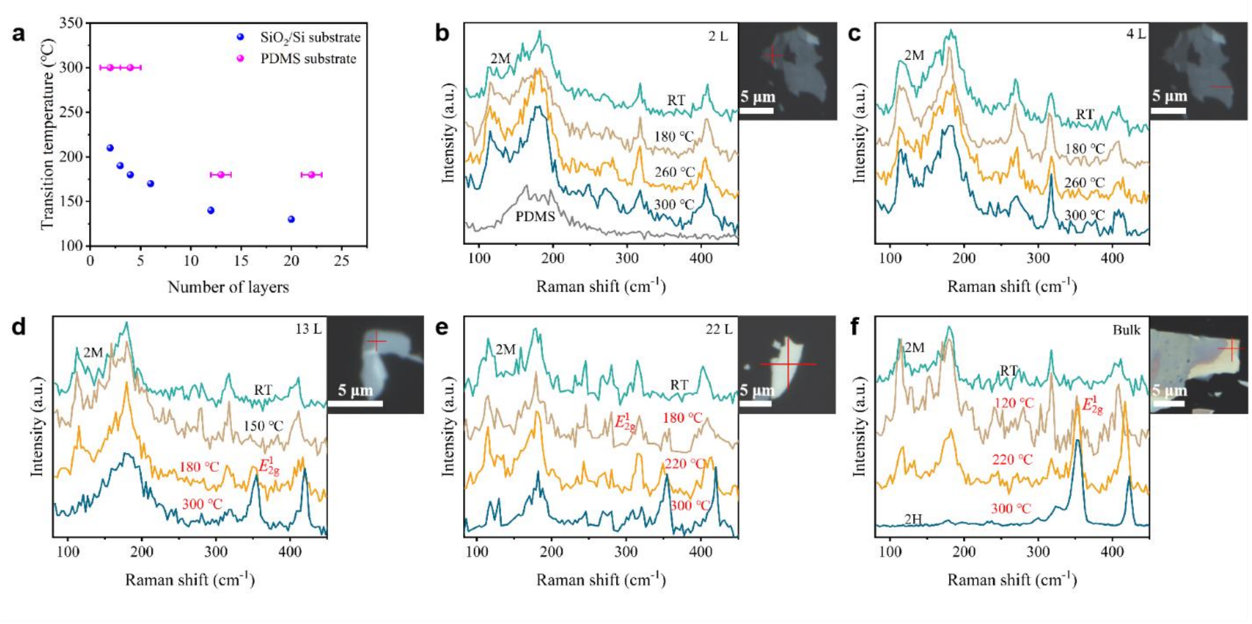

Supplementary Fig. 13. (a) 2M to 2H phase transition temperatures as a function of WS_2 layer thickness measured at PMDS and SiO_2/Si substrates in the air. In the heating program, temperature was elevated by 5 °C and held for 1 min in each step. RT is short of room temperature. (b-f) Optical images at room temperature and Raman spectra of 2M WS_2 flakes with different thicknesses on PDMS substrates at different temperatures. Activation of 2M to 2H phase transition is indicated by emergence of the E^1_{2g} mode at 350.2 cm^{-1}.

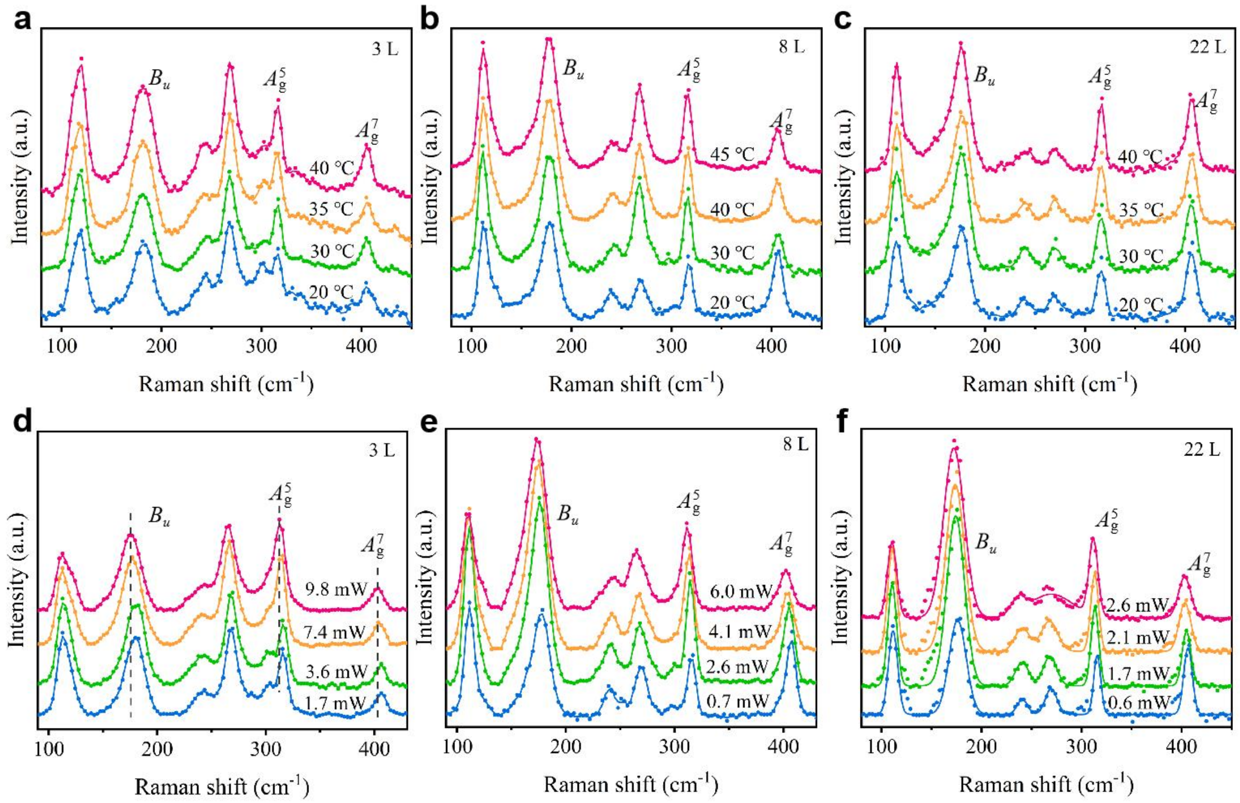

Supplementary Fig. 14. Raman spectra of a 3 L, an 8 L and a 22 L 2M WS_2 acquired (a-c) at different temperatures and (d-f) with different-power laser excitation.

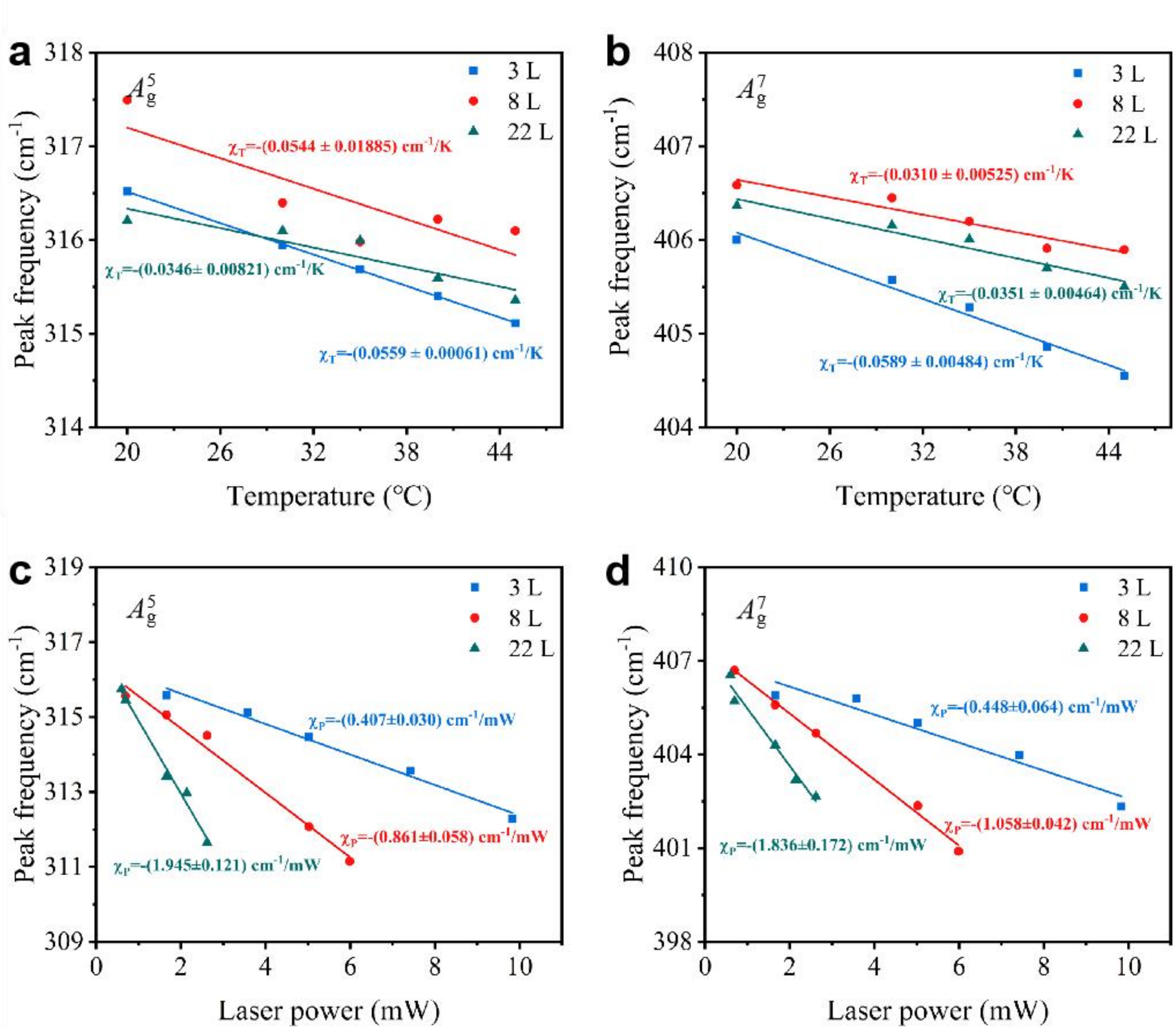

Supplementary Fig. 15. (a-b) Frequency of A_g^5 and A_g^7 Raman modes as functions of temperature and (c-d) frequency of A_g^5 and A_g^7 Raman modes as functions of laser power for 3 L, 8 L and 22 L 2M WS_2. Temperature coefficient (χ_T) and laser-power coefficient (χ_P) of Raman frequencies are extracted from the slopes of the corresponding plots.

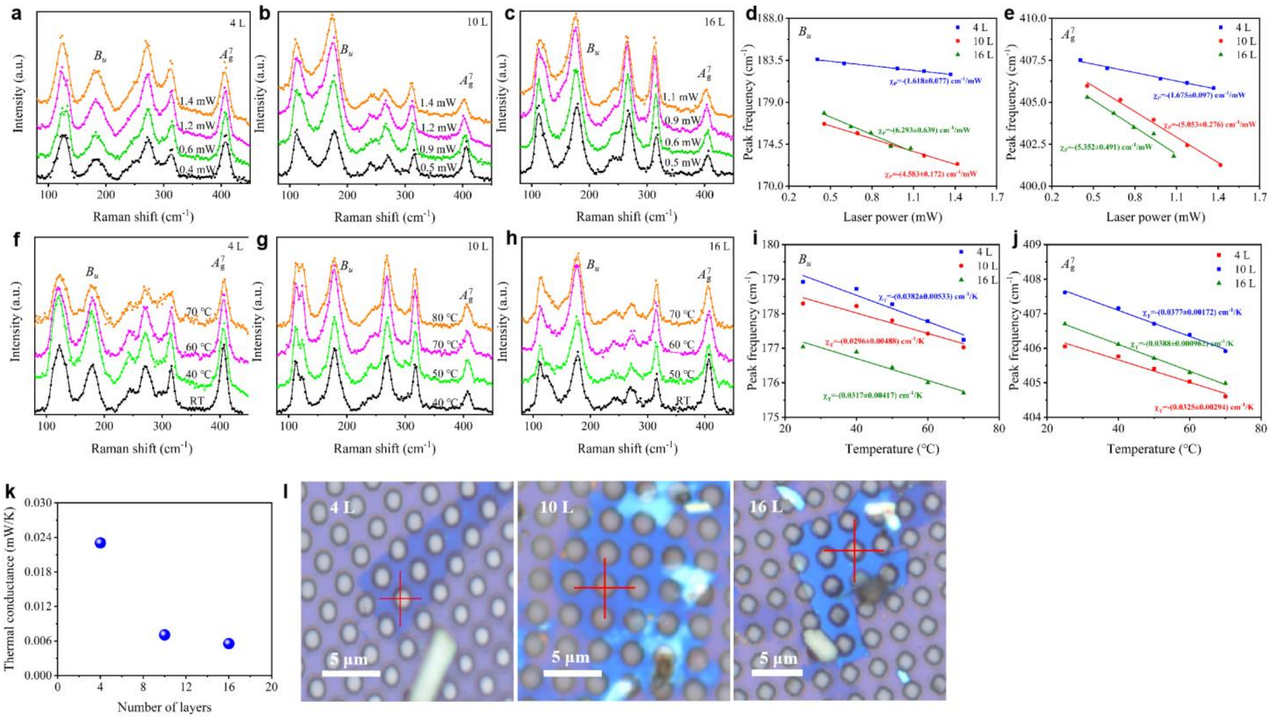

Supplementary Fig. 16. Thickness-dependent thermal conductivity of 2M WS_2 on holey Si/SiO_2 substrate. Raman spectra of (a) 4 L, (b) 10 L and (c) 16 L 2M WS_2 acquired with different-power laser excitation. Frequency of (d) B_u and (e) A_g^7 Raman modes as functions of laser power for 4 L, 10 L and 16 L 2M WS_2. Laser-power coefficient (χ_P) of Raman frequencies are extracted from the slopes of the corresponding plots. Raman spectra of (f) 4 L, (g) 10 L and (h) 16 L 2M WS_2 acquired at different temperatures. Frequency of (i) B_u and (j) A_g^7 Raman modes as functions of temperature for 4 L, 10 L and 16 L 2M WS_2. Temperature coefficient (χ_T) of Raman frequencies are extracted from the slopes of the corresponding plots. (h) Thermal conductivities of 4 L, 10 L and 16 L 2M WS_2 estimated by χ_T divided by χ_P. For each sample, an average thermal conductivity is obtained based on two values calculated from three sets of χ_T and χ_P data that are extracted from B_u and A_g^7 modes plots.

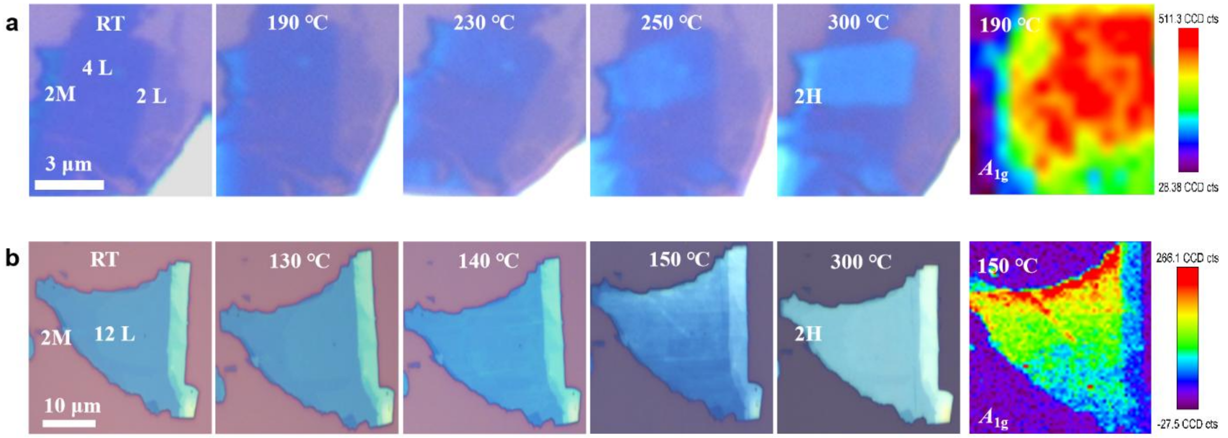

Supplementary Fig. 17. Optical images and E^1_{2g} Raman mode mappings of a piece of (a) 2 ~ 4 L and (b) 12 L 2M WS_2 at different temperatures in the air. In the heating program, temperature was elevated by 5 °C and held for 1 min in each step. RT is short of room temperature. The phase change area in the WS_2 flake turns opaque, since 2H WS_2 has a higher infraction index than 2M WS_2. 12 L WS_2 shows 2M/2H stripes patterns during the heating program, but the 2 ~ 4 L WS_2 does not show this pattern.

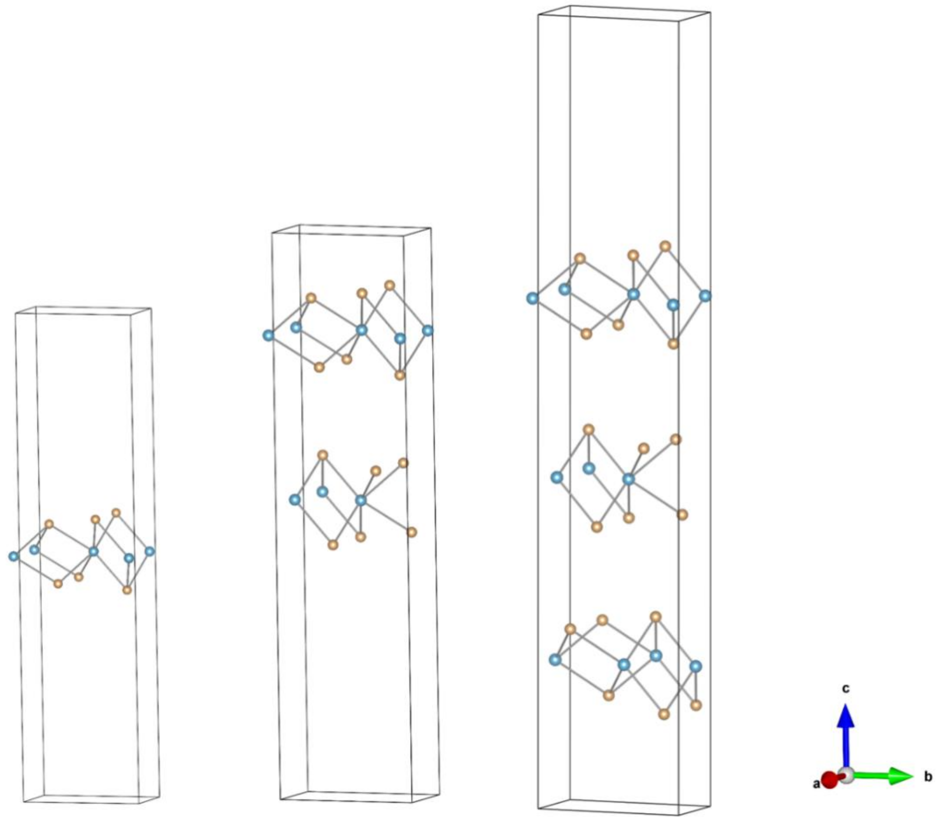

Supplementary Fig. 18. Structures of initial state rectangular 1 L (left), and 2 L (middle) and 3 L (right) WS_2 supercells applied in theoretical calculations. 1 L, 2 L and 3 L WS_2 supercells are defined to contain one, two and three 1T' layers, respectively. Color code: blue and orange spheres represent W and S, respectively.

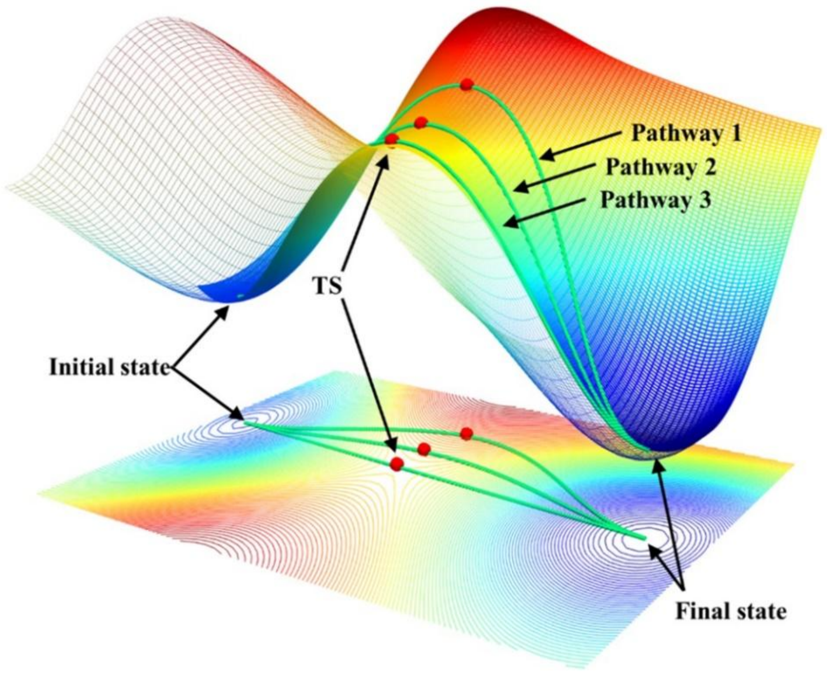

Supplementary Fig. 19. Illustration of different pathways connecting the initial state and the final state on a potential energy surface (PES), and the connection pathways and the corresponding energy maximum (EM) points are plotted in green lines and red dots, respectively. The EM point of pathway 3 has the lowest energy among all EM points of connection pathways, corresponding to the exact transition state (TS) connecting the initial state and the final state. Reprinted with permission from Supplementary ref. 2. Copyright 2022 American Chemical Society.

1 L WS_2

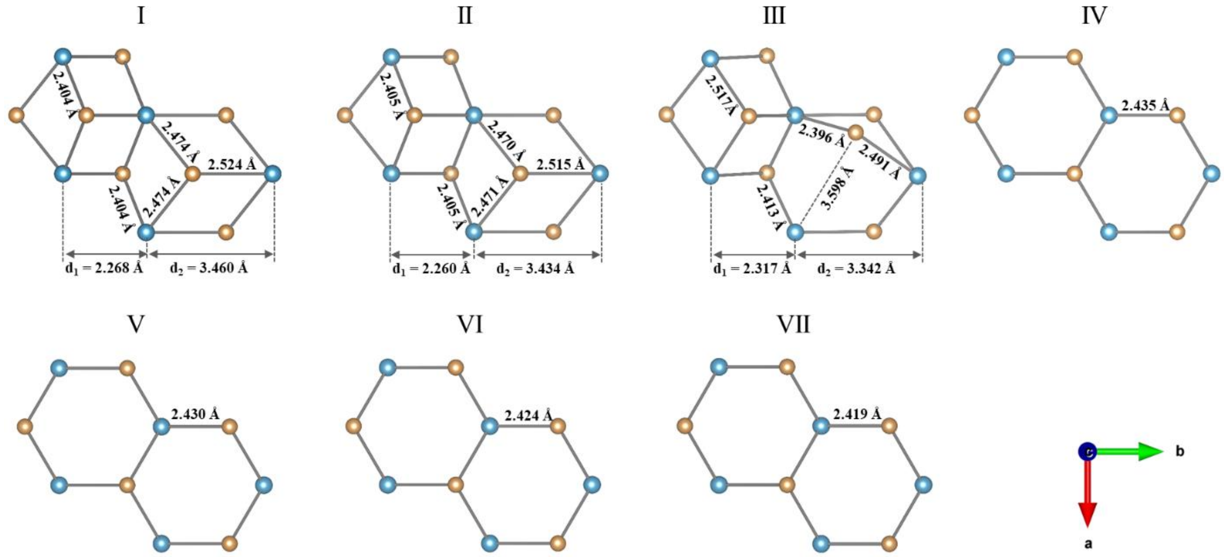

Supplementary Fig. 20. The molecular geometries of 1 L WS_2 supercell at the initial 1T′ phase (coord. I), transition state (coord. III), final 1H phase (coord. VII) and other intermediate configurations, viewing from the *c* direction. Color code: blue and orange spheres represent W and S, respectively.

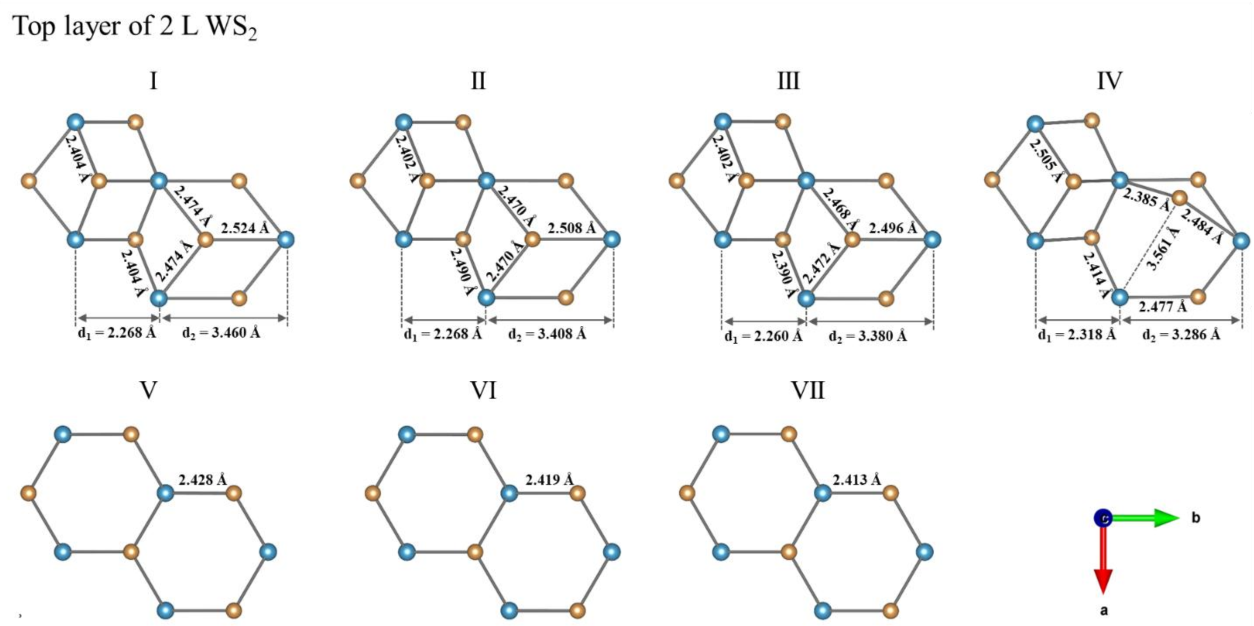

Supplementary Fig. 21. The molecular geometries of top layer of 2 L WS_2 supercell at the initial 1T′ phase (coord. I), transition state (coord. IV), final 1H phase (coord. VII) and other intermediate configurations, viewing from the *c* direction. Color code: blue and orange spheres represent W and S, respectively.

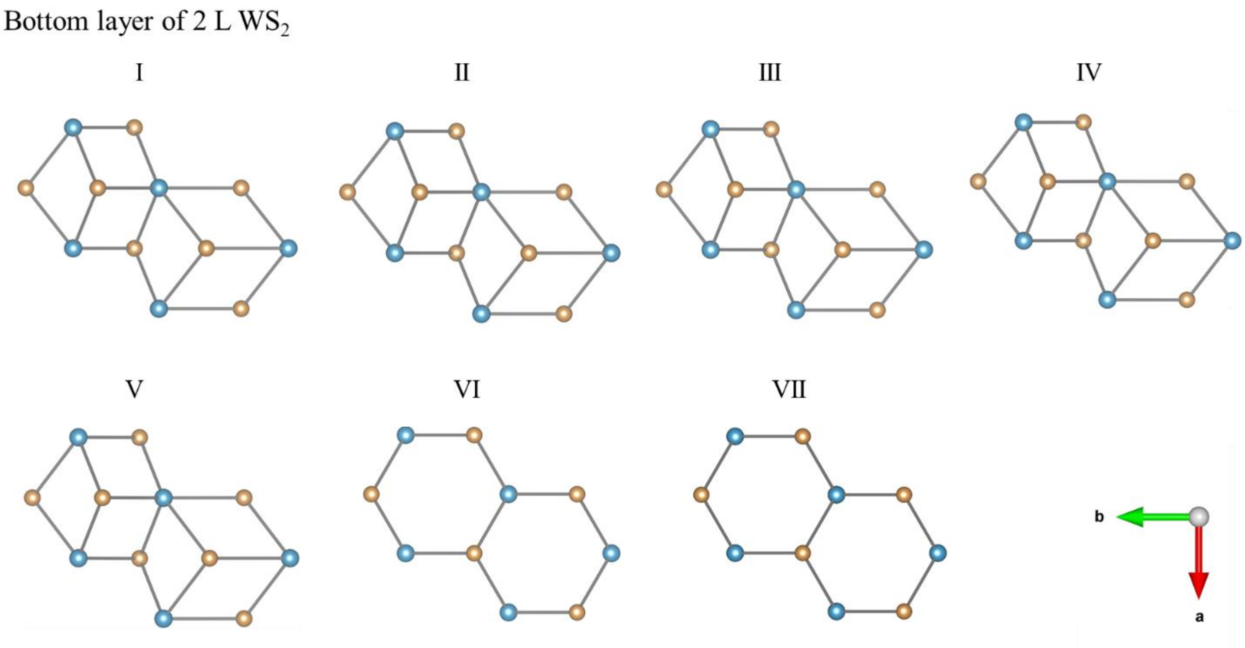

Supplementary Fig. 22. The molecular geometries of bottom layer of 2 L WS_2 supercell at the initial 1T′ phase (coord. I), transition state (coord. IV), final 1H phase (coord. VII) and other intermediate configurations, viewing from the *c* direction. Color code: blue and orange spheres represent W and S, respectively.

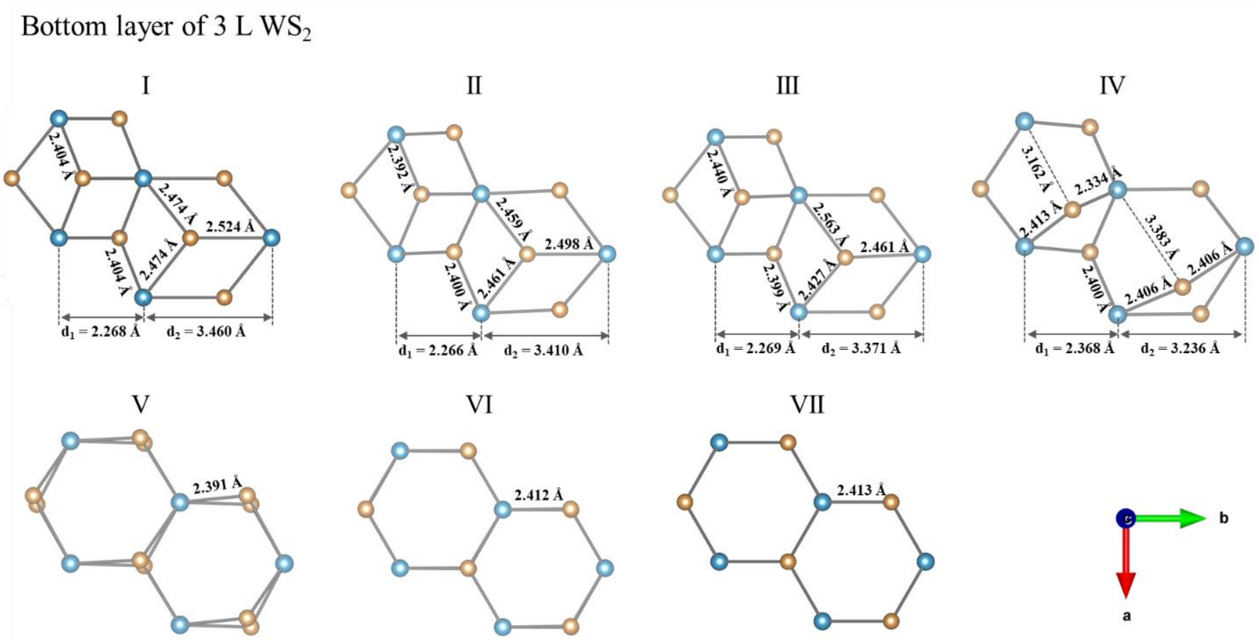

Supplementary Fig. 23. The molecular geometries of bottom layer of 3 L WS_2 supercell at the initial 1T′ phase (coord. I), transition state (coord. III), final 1H phase (coord. VII) and other intermediate configurations, viewing from the *c* direction. Color code: blue and orange spheres represent W and S, respectively.

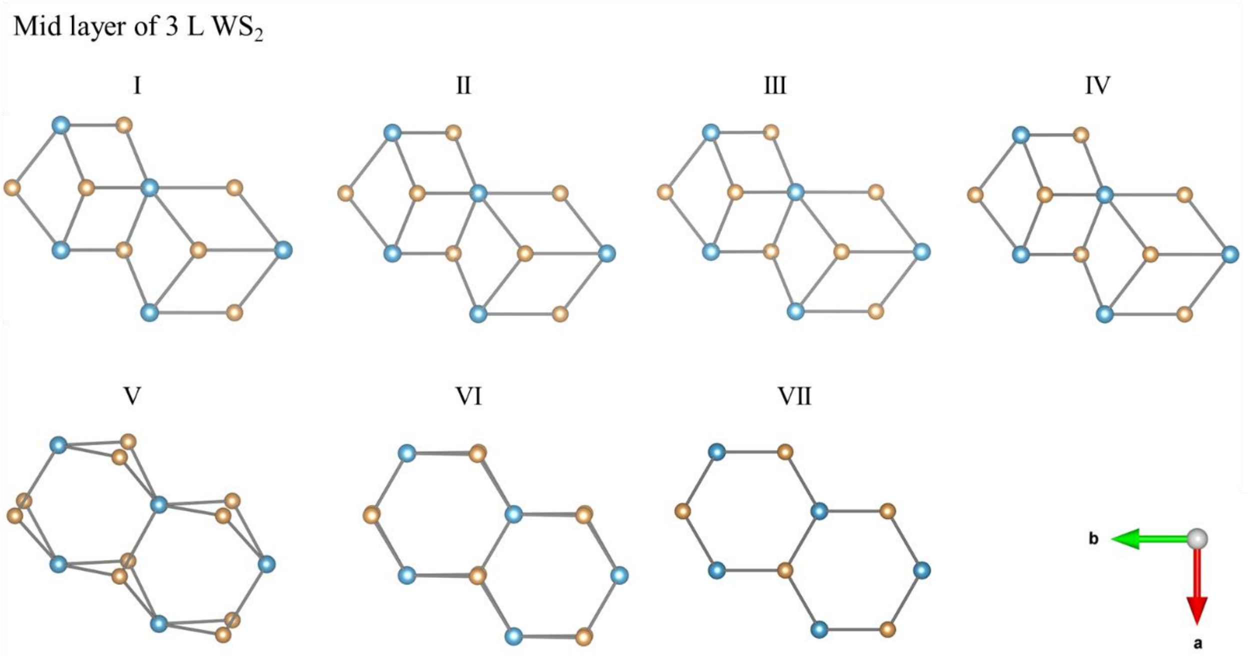

Supplementary Fig. 24. The molecular geometries of mid layer of 3 L WS_2 supercell at the initial 1T′ phase (coord. I), transition state (coord. III), final 1H phase (coord. VII) and other intermediate configurations, viewing from the *c* direction. Color code: blue and orange spheres represent W and S, respectively.

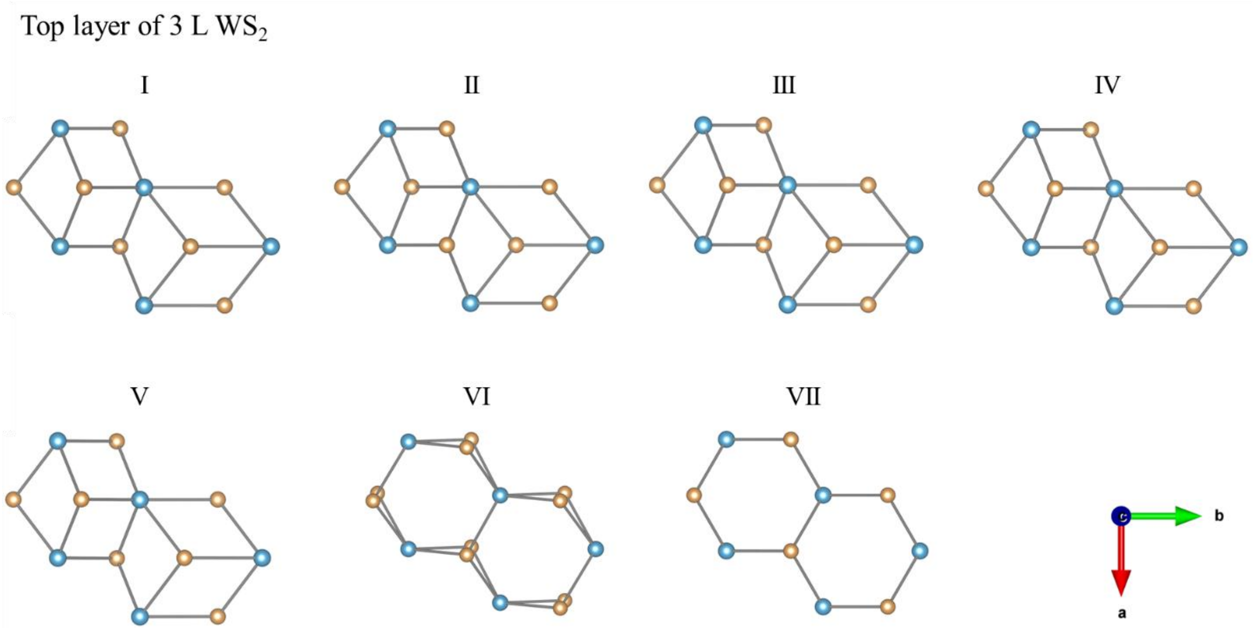

Supplementary Fig. 25. The molecular geometries of mid layer of 3 L WS_2 supercell at the initial 1T′ phase (coord. I), transition state (coord. III), final 1H phase (coord. VII) and other intermediate configurations, viewing from the *c* direction. Color code: blue and orange spheres represent W and S, respectively.

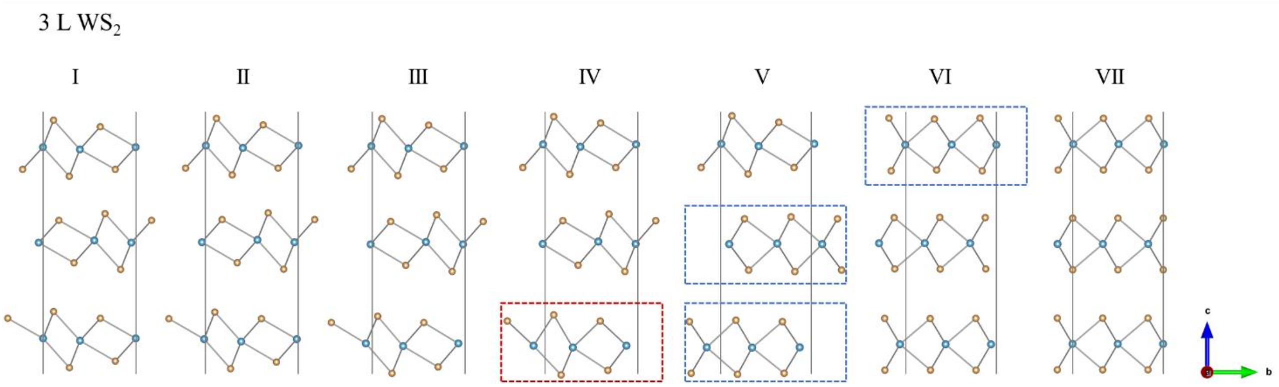

Supplementary Fig. 26. The molecular geometries of 3 L WS_2 supercell at the initial phase state (coord. I), transition state (coord. IV), final phase state (coord. VII) and other intermediate configurations, viewing from the *a* direction. The highly deformed layer at the transition state is marked by a red dotted square. Layers turned from 1T′ to 1H types of structures are marked by blue dotted squares. Lattice edges are depicted in different states, referring to which interlayer dislocations can be seen. Color code: blue and orange spheres represent W and S, respectively.

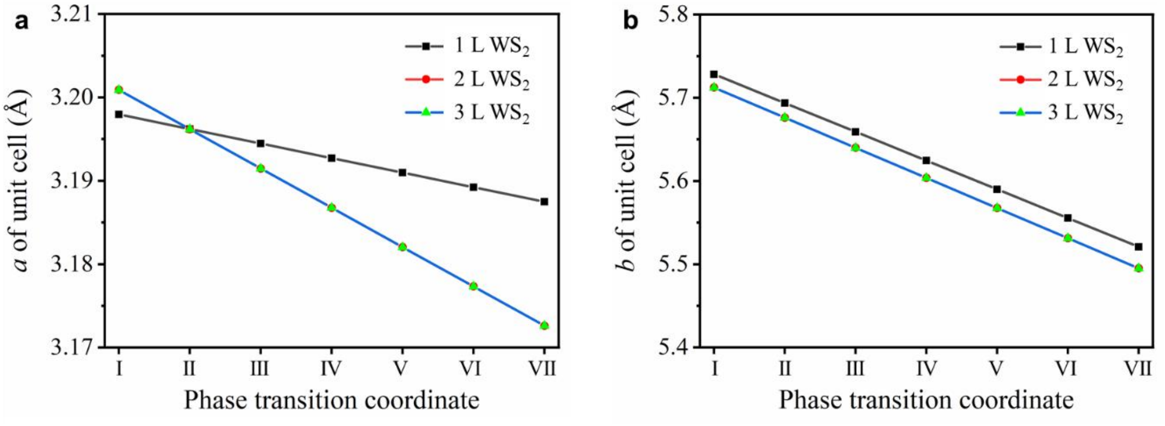

Supplementary Fig. 27. Variation of calculated WS_2 unit cell parameter of (a) *a* and (b) *b* in the initial 2M phase (I), final 2H phase (VII), transition state and other intermediate configurations (II to VI), showing lattice shrinkages during 2M to 2H phase transition.

Supplementary Table 2. Molecular geometries WS_2 layers at the initial 1T′ phase state and the transition states in the 1 L, 2 L and WS_2 supercells. Highly deformed layers at the transition states are marked by red dotted squares.

<table>
<tr><th>Initial state</th><th colspan="3">Transition state</th></tr>
<tr><th>1T′</th><th>1 L WS_2</th><th>2 L WS_2</th><th>3 L WS_2</th></tr>
<tr>
<td>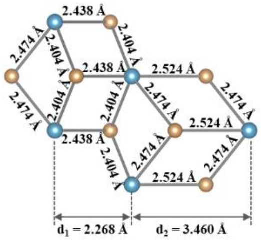

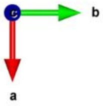
</td>
<td>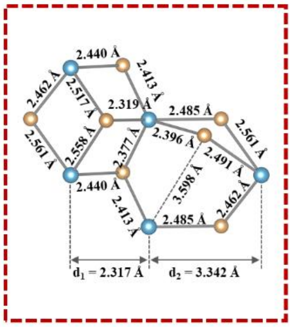
</td>
<td>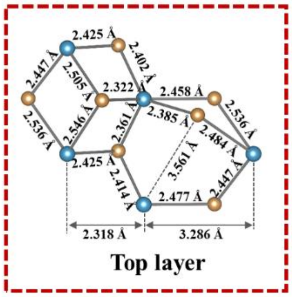

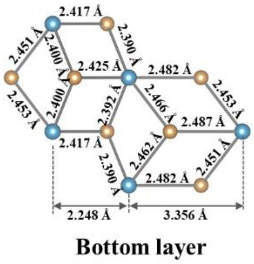
</td>
<td>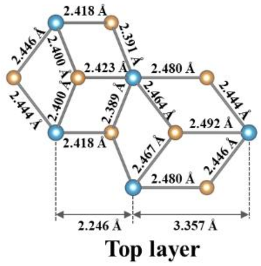

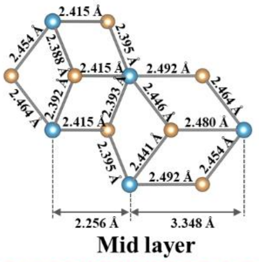

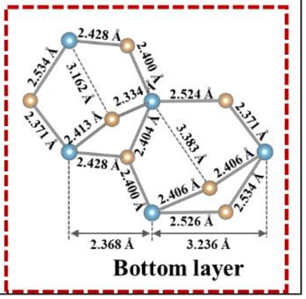
</td>
</tr>
</table>

V. Supplementary references

1. Fang, Y. *et al.* Discovery of superconductivity in 2M WS_2 with possible topological surface states. *Advanced Materials* **31**(2019).

2. Liu, Y., Qi, H. & Lei, M. Elastic Image Pair Method for Finding Transition States on Potential Energy Surfaces Using Only First Derivatives. *Journal of Chemical Theory and Computation* **18**, 5108-5115 (2022).

3. Henkelman, G., Uberuaga, B.P. & Jonsson, H. A climbing image nudged elastic band method for finding saddle points and minimum energy paths. *Journal of Chemical Physics* **113**, 9901-9904 (2000).

4. Kresse, G. & Furthmuller, J. Efficient iterative schemes for ab initio total-energy calculations using a plane-wave basis set. *Physical Review B* **54**, 11169-11186 (1996).

5. Kresse, G. & Joubert, D. From ultrasoft pseudopotentials to the projector augmented-wave method. *Physical Review B* **59**, 1758-1775 (1999).

6. Perdew, J.P., Burke, K. & Ernzerhof, M. Generalized gradient approximation made simple. *Physical Review Letters* **77**, 3865-3868 (1996).

7. Pack, J.D. & Monkhorst, H.J. Special points for Brillouin-zone integrations. *Physical Review B* **16**, 1748-1749 (1977).

8. Grimme, S. Semiempirical GGA-type density functional constructed with a long-range dispersion correction. *Journal of Computational Chemistry* **27**, 1787-1799 (2006).

9. Schutte, W.J., Deboer, J.L. & Jellinek, F. Crystal structures of tungsten disulfide and diselenide. *Journal of Solid State Chemistry* **70**, 207-209 (1987).
