# Supplementary material for: High intrinsic phase stability of ultrathin 2M WS2
Source: Nat Commun. 2024 Feb 10;15:1263. doi: 10.1038/s41467-024-45676-3 (PMC10858873; doi:10.1038/s41467-024-45676-3)
Supplement: Supplementary file 3 — Description of Additional Supplementary Files [file 41467_2024_45676_MOESM3_ESM.pdf]

Description of Additional Supplementary Files

File Name: Supplementary Software 1
Description: Calculated structures of WS_2 supercells.
